# A Novel Prokaryote-Type ECF/ABC Transporter Module in Chloroplast Metal Homeostasis

**DOI:** 10.3389/fpls.2019.01264

**Published:** 2019-10-29

**Authors:** Lena Voith von Voithenberg, Jiyoung Park, Roland Stübe, Christopher Lux, Youngsook Lee, Katrin Philippar

**Affiliations:** ^1^Plant Biochemistry and Physiology, Department of Biology I, LMU München, Planegg-Martinsried, Germany; ^2^Department of Life Science, Pohang University of Science and Technology, Pohang, South Korea; ^3^Plant Biology, Center for Human and Molecular Biology (ZHMB), Saarland University, Saarbrücken, Germany

**Keywords:** ABC transporter, chloroplast, energy-coupling factor transporter, inner envelope membrane, iron transport, metal homeostasis

## Abstract

During evolution, chloroplasts, which originated by endosymbiosis of a prokaryotic ancestor of today’s cyanobacteria with a eukaryotic host cell, were established as the site for photosynthesis. Therefore, chloroplast organelles are loaded with transition metals including iron, copper, and manganese, which are essential for photosynthetic electron transport due to their redox capacity. Although transport, storage, and cofactor-assembly of metal ions in chloroplasts are tightly controlled and crucial throughout plant growth and development, knowledge on the molecular nature of chloroplast metal-transport proteins is still fragmentary. Here, we characterized the soluble, ATP-binding ABC-transporter subunits ABCI10 and ABCI11 in *Arabidopsis thaliana*, which show similarities to components of prokaryotic, multisubunit ABC transporters. Both ABCI10 and ABCI11 proteins appear to be strongly attached to chloroplast-intrinsic membranes, most likely inner envelopes for ABCI10 and possibly plastoglobuli for ABCI11. Loss of ABCI10 and ABCI11 gene products in *Arabidopsis* leads to extremely dwarfed, albino plants showing impaired chloroplast biogenesis and deregulated metal homeostasis. Further, we identified the membrane-intrinsic protein ABCI12 as potential interaction partner for ABCI10 in the inner envelope. Our results suggest that ABCI12 inserts into the chloroplast inner envelope membrane most likely with five predicted α-helical transmembrane domains and represents the membrane-intrinsic subunit of a prokaryotic-type, energy-coupling factor (ECF) ABC-transporter complex. In bacteria, these multisubunit ECF importers are widely distributed for the uptake of nickel and cobalt metal ions as well as for import of vitamins and several other metabolites. Therefore, we propose that ABCI10 (as the ATPase A-subunit) and ABCI12 (as the membrane-intrinsic, energy-coupling T-subunit) are part of a novel, chloroplast envelope-localized, AAT energy-coupling module of a prokaryotic-type ECF transporter, most likely involved in metal ion uptake.

## Introduction

Chloroplasts originated about 3 billion years ago by endosymbiosis of an ancestor of today’s cyanobacteria with a mitochondria-containing host cell (Gould et al., 2008; Zimorski et al., 2014). During evolution, chloroplasts were established as the site for photosynthesis and thus became the basis for all life dependent on oxygen and carbohydrate supply. To fulfill this task, chloroplast organelles are loaded with the transition metals iron (Fe), copper (Cu), and manganese (Mn), which are essential for photosynthetic electron transport due to their redox capacity (Yruela, 2013). In consequence, chloroplasts represent the Fe-richest system in plant cells (Raven et al., 1999). However, evolutionary improvement of oxygenic photosynthesis in turn required tight control of metal transport and distribution since metal-catalyzed generation of reactive oxygen species (ROS) causes oxidative damage. This is most acute in chloroplasts, where oxygen radicals and transition metals are side by side and ROS-production is a usual feature of photosynthetic electron transport (Asada, 1999; Mubarakshina et al., 2010). Thus, on the one hand, when chelated or bound by proteins, chloroplast-intrinsic metals are a prerequisite for photoautotrophic life, but on the other hand become toxic when present in their highly reactive, radical generating, free ionic forms (Briat et al., 2010; Halliwell and Gutteridge, 1992). In consequence, transport, storage and cofactor-assembly of metal ions in chloroplasts have to be tightly controlled and are crucial throughout plant growth and development [for overview see (Thomine and Vert, 2013; Bashir et al., 2016; Lopez-Millan et al., 2016; Vigani et al., 2019)].

Due to their endosymbiotic origin, chloroplasts are surrounded by two membranes similar to their Gram-negative prokaryotic ancestors. Whereas the inner envelope (IE) membrane of chloroplasts presumably was derived mainly from the bacterial plasma membrane, the chloroplast outer envelope (OE), however, largely originated from the outer membrane of the Gram-negative cyanobacterial-like endosymbiont (Block et al., 2007). In the IE, numerous transporter proteins for metabolites and ions have been characterized (Weber and Linka, 2011; Finazzi et al., 2015; Marchand et al., 2018). These channels and transporters are facilitating the exchange of ions and metabolic products between plastids and the cytoplasm. Transmembrane-spanning domains of these proteins are built by hydrophobic α-helices. In contrast, characteristic channels of the outer membrane in Gram-negative bacteria and the chloroplast OE span the membrane in the form of β-strands that are organized to form a barrel-like pore structure (Duy et al., 2007a; Zeth and Thein, 2010). In chloroplasts, these solute pores like the OE proteins OEP21, OEP24, OEP37 and OEP40 (Pohlmeyer et al., 1998; Bölter et al., 1999; Goetze et al., 2006; Harsman et al., 2016) are essential parts of the outer membrane permeom for metabolites and ions (Breuers et al., 2011).

The metal transport mechanisms of chloroplasts (Lopez-Millan et al., 2016; Nouet et al., 2011; Vigani et al., 2019) are not as well-known as strategies occurring in root plasma membranes for iron acquisition, i.e., reduction-based Fe^2+^-transport (strategy I) and transport of chelated Fe^III^ [strategy II; see (Morrissey and Guerinot, 2009; Kobayashi and Nishizawa, 2012; Brumbarova et al., 2015)]. However, research on the molecular identity of chloroplast iron transport systems suggests that several protein families may play a role in Fe-uptake and export [for overview see (Nouet et al., 2011; Finazzi et al., 2015; Lopez-Millan et al., 2016)]. Chloroplasts inherited a series of solute transporters from their prokaryotic ancestors (Tyra et al., 2007), including components of metal transport systems like the ancient, Fe-uptake permease PIC1 of cyanobacterial origin (Duy et al., 2007b) or the prokaryotic-type ATP-binding cassette (ABC) transporter subunit ABCI11/NAP14 (Shimoni-Shor et al., 2010). The permease PIC1 previously also has been described as Tic21, a putative IE translocon component, which could participate in import of nuclear-encoded plastid proteins from the cytosol (Teng et al., 2006). However, a direct functional analysis of Tic21/PIC1 for protein transport is lacking. In 2009, a protein complex of about 1 MDa was identified at the chloroplast IE membrane containing the putative translocon channel Tic20, a large fraction (about 900 kDa) of yet unidentified membrane proteins, and also small amounts of Tic21/PIC1 (Kikuchi et al., 2009). More recent publications, however, which lead to the identification of the other proteins in this potential protein translocation core, demonstrated that Tic21/PIC1 does not co-purify with this 1 MDa complex (Kikuchi et al., 2013; Nakai, 2015). Therefore, the previously described function of PIC1 in protein import seems to be obsolete [for discussion see (Duy et al., 2007b; Duy et al., 2011; Lopez-Millan et al., 2016)]. Further, transporters of prokaryotic origin are involved in the shuttling of Mn across the IE and thylakoid membranes in chloroplasts (Eisenhut et al., 2018; Krieger-Liszkay and Thomine, 2018; Zhang et al., 2018) as well as membranes of the cyanobacterium *Synecocystis PCC6803* (Brandenburg et al., 2017; Gandini et al., 2017). Therefore, metal transport mechanisms of prokaryotes, in particular those of Gram-negative bacteria and cyanobacteria (Braun and Hantke, 2011; Lau et al., 2016), today can serve as blueprint for chloroplasts of land plants. Proteins that transport metals across the OE have not been identified yet nevertheless they might be represented by ß-barrel channel pores like OEPs or the protein translocon channel Toc75. In Gram-negative *E. coli* and also in cyanobacteria, Fe uptake across the OM occurs *via* receptor-gated ß-barrel channels also called TonB-dependent transporters (TBDTs), which transport Fe^III^-chelates and are energized by the TonB system at the plasma membrane (Kranzler et al., 2013; Braun, 2014; Rudolf et al., 2015). Whereas physiological data point to a reduction based transport of divalent Fe^2+^ or Mn^2+^ for metal uptake across the IE membrane (Shingles et al., 2001; Shingles et al., 2002; Solti et al., 2012; Solti et al., 2014), chelated iron most likely in the form of Fe^III^-citrate complexes is shuttled over the OE membrane (Bughio et al., 1997; Solti et al., 2012; Solti et al., 2016; Müller et al., 2018). For a recent update on intracellular iron transport in plants, we refer to Vigani et al. (2019).

In plants, an exceptionally high number of ABC transporters exists, which are involved in the transport and distribution of numerous metabolites and ions, including metals, hormones, and lipid compounds. In consequence, functional ABC-transporter systems are crucial for plant growth and development (Do et al., 2018; Hwang et al., 2016). The classical, eukaryotic ABC transporters are composed of two nucleotide binding (NBD) and two transmembrane (TMD) permease subunits. Depending if these four subunits are encoded by one or two genes, the proteins are categorized as full- or half-size ABC transporters, respectively (Verrier et al., 2008; Theodoulou and Kerr, 2015). For functional half-size transporters, a homo- or heterodimer of NBD-TMD or TMD-NBD proteins can be assembled. In addition to these eukaryotic ABC-transporters sorted into subfamilies A-D and G, plants possess a collection of ABC proteins bearing similarities to components of prokaryotic, multisubunit ABC transporters (Verrier et al., 2008; Theodoulou and Kerr, 2015). In general, canonical, prokaryotic ABC transporters as well assemble as dimers of two NBD and two TMD proteins, however, for importers, an additional substrate binding protein (SBP) exists (Eitinger et al., 2011; Theodoulou and Kerr, 2015). In contrast to the operon arrangement of prokaryotes, however, in plants, separate intron-containing genes, which are scattered throughout the genome, encode for the subunits of these transporters. Thereby, the correct identification of the single subunits that form one functional ABC complex is hindered. In the first inventory of plant ABC transporters, several of the prokaryotic-type, soluble NBD-subunits were designated as non-intrinsic ABC proteins (NAPs) (Sanchez-Fernandez et al., 2001). However, later on, most of the NAPs were grouped into ABC protein subfamily I (Verrier et al., 2008). Well in line with their prokaryotic features, most of these *Arabidopsis* ABCI proteins characterized so far are localized in the endosymbiotically derived organelles chloroplasts and mitochondria. These include ABCI1/NAP10 (NBD), ABCI2 (TMD) for the cytochrome *c* maturation complex in mitochondria (Rayapuram et al., 2007), and ABCI6/NAP7 (NBD), ABCI7, and ABCI8 for the iron-sulfur cluster biogenesis complex in chloroplasts (Xu and Möller, 2004). The well characterized TGD1/ABCI4 (TMD), TGD2/ABCI5 (SBP), and TGD3/ABCI13 (NBD) ABC transporter complex in the chloroplast IE is responsible for the import of eukaryotic acyl lipids, synthesized in the ER (Xu et al., 2003; Lu et al., 2007; Xu et al., 2010; Roston et al., 2012). Further, the NBD protein ABCI17/NAP3 was described to bind to ABCI16/ALS3 (TMD) in the tonoplast and to be implicated in root metal homeostasis (Fe, Al) and signaling under phosphate deficiency (Huang et al., 2010; Belal et al., 2015; Dong et al., 2017; Wang et al., 2019). Only for the TGD complex, a distinct membrane transport function, i.e., lipid import into chloroplasts, is described. With its NBD-TMD-SBP subunit arrangement, TGD1-3 corresponds to the full canonical ABC importer assembly in prokaryotes. ABCI1-ABCI2 (cytC maturation in mitochondria) and ABCI17-ABCI16 (root metal homeostasis, signaling at phosphate deficiency) are supposed to assemble as NBD-TMD dimers. The NBD ATPase ABCI6 for FeS cluster biogenesis in chloroplasts, however, interacts with ABCI7 and ABCI8, which are soluble proteins that do not belong to an ABC transporter assembly. The chloroplast intrinsic NBD-subunit ABCI11/NAP14 in *Arabidopsis* and rice (here designated as Os-ABCI8) was described to play a pivotal role in metal homeostasis, although a direct involvement in transport or interaction with membrane-intrinsic ABC transporter subunits so far has not been demonstrated (Shimoni-Shor et al., 2010; Zeng et al., 2017).

In addition to the canonical NBD-TMD-SBP ABC-complexes for metabolite and ion uptake, prokaryotes contain a differently organized class of importing ABC proteins, also known as energy-coupling factor (ECF) transporters (Rodionov et al., 2009; Eitinger et al., 2011; Theodoulou and Kerr, 2015; Rempel et al., 2019). Central to these ECF protein complexes is the energy-coupling module AAT, which consists of two NBD ATPase-subunits (A) and one energy-transducing, transmembrane subunit T. The A subunits contain the classical ATPase and ABC transporter signature motifs within the RecA and helical subdomains (Davidson et al., 2008; Wilkens, 2015). Both A proteins bind to a single T protein either as homodimer (A1, A1), heterodimer (A1, A2), or a pair of ATPase domains fused in a single polypeptide (A1-A2) (Rodionov et al., 2009; Eitinger et al., 2011). In contrast to the canonical prokaryotic ABC importers (see above), the substrate-binding subunit of ECF transporters (S) is a membrane-intrinsic protein. ECF-type ABC importers are divided into two subgroups, depending if they contain a dedicated energy-coupling module for each S subunit (group I) or a shared AAT module that can be combined with various substrate-binding S components (group II) (Rodionov et al., 2009; Eitinger et al., 2011; Rempel et al., 2019). Besides several other metabolites, group I ECF transporters in bacteria are described to transport biotin (BioMNY complex; Hebbeln et al., 2007) and are widely distributed for the uptake of cobalt and nickel metal ions *via* CbiMNQO and NikMNQO complexes, respectively (Rodionov et al., 2006). Because of the high substrate variability of the different S components, which form complexes with an invariant EcfAAT module, group II ECF transporters have a broad range of substrate metabolites, including folate (FolT transporter; Neubauer et al., 2009; Rodionov et al., 2009) as well as other vitamins, metabolites, cofactors, and precursors thereof (Eitinger et al., 2011).

Currently, five crystal structures of full group II complexes and one structure of a group I ECF transporter are resolved (Rempel et al., 2019). Among them, the CbiMQO core complex (group I, EcfAATS) from *Rhodobacter capsulatus* (Bao et al., 2017) and the structure of FolT2 (group II, EcfA1A2TS) from *Lactobacillus delbrueckii* (Swier et al., 2016) allow insight into the molecular mechanism for divalent metal and folate transport, respectively (Rempel et al., 2019). The ATPase subunits (A components) contain all domains (RecA, helical subdomains) and motifs described for ABC transporter NBD subunits (Davidson et al., 2008; Wilkens, 2015). In addition, ECF transporter ATPases are characterized by a special arrangement of the negatively charged groove that exists on the surface of the AA dimer. This groove, which is formed by the so-called Q-helix and the first helix of the helical subdomain of each A subunit, is responsible for contact with the T subunit (Karpowich and Wang, 2013; Rempel et al., 2019). The Q-helix is specific for polypeptide chains of ECF transporter ATPases and forms a highly conserved, short helical turn with a conserved amino acid motif. This six-residue helix, named after the invariant glutamine (Q), allows correct positioning of a conserved acidic amino acid from the helical subdomain within the groove of the AA dimer. Thereby, this acidic and negatively charged residue of each A subunit is enabled to bind to the strictly conserved and positively charged arginine (X-R-X motif) in one of the two coupling helices of the membrane-intrinsic T subunit (Swier et al., 2016; Bao et al., 2017; Rempel et al., 2019). Furthermore, a conserved negatively charged amino acid (aspartate or glutamate) of the Q-helix itself forms an intramolecular interaction with a specific arginine of the “LSGGQ” motif in the helical subdomain of each A component (Karpowich and Wang, 2013). Thereby, the Q-helix is essential for transport activity of ECF transporters and significantly contributes to the strong interactions described between ATPase and T subunits of prokaryotic ECF importers (Bao et al., 2017; Rempel et al., 2019). ATPase proteins of group II ECF transporters further are characterized by short, C-terminal helices, which contribute to dimerization and are absent in many ATPases of group I ECF transporters (Rodionov et al., 2009; Karpowich and Wang, 2013). T subunits of different ECF transporters contain a varying amount of α-helical transmembrane domains (between 4 and 8) and the coupling domain for contact with both A proteins. This coupling domain comprises two long α-helices that are arranged in an X shape at the cytosolic face of the membrane (Bao et al., 2017; Rempel et al., 2019). At the C-terminal end of each of these coupling helices, a conserved arginine motif (X-R-X, mostly Ala-Arg-Gly) (Eitinger et al., 2011) mediates interaction with the groove of the AA dimer (see above) and is essential for ECF complex assembly as well as ATPase activity (Neubauer et al., 2009). Transport by ECF importers is energized by ATP hydrolysis *via* the AA dimer, which in turn most likely induces a swing like movement of the transmembrane helices of subunit T [for details on mechanisms see (Bao et al., 2017; Rempel et al., 2019)]. Substrate specificity of each transporter complex is defined by the S components, which have a core of six α-helical membrane domains arranged in a cylindrical bundle. The variable substrate binding side is located in a pocket at the extracellular side of the membrane (Rempel et al., 2019). Group I S components for Ni and Co metal ECF transporters—i.e., NikM and CbiM—contain an additional, N-terminal α-helix that is involved in substrate binding with its first two amino acids. In addition, these metal ion ECF transporter complexes assemble with a third, short membrane-spanning domain (two α-helices)—i.e., subunit N in NikMNQO and CbiMNQO (Eitinger et al., 2011).

In eukaryotes, assembly of ECF transporter subunits, however, has never been described before. A plant-specific T subunit, predicted to be targeted to chloroplasts and corresponding to cyanobacterial T proteins, was identified by *in silico* analyses (Rodionov et al., 2009; Eitinger et al., 2011). However, the corresponding ATPase and substrate binding subunits remain elusive. Only in the chloroplast genome of the freshwater green algae *Mesostigma viride*, a single BioY ortholog (S-component) was discovered (Hebbeln et al., 2007). Due to their endosymbiotic origin and high metal content, chloroplasts represent the most probable site for metal-transporting ECF proteins. Furthermore, chloroplast import of metabolites is essential for cellular metabolism, e.g., biotin uptake for plastid-intrinsic *de novo* fatty acid biosynthesis. Our results on the chloroplast-localized ABCI proteins ABCI10 and ABCI12 here for the first time point to such an ECF ABC-transporter core complex in the IE membrane of chloroplasts. Therefore, we propose that ABCI10 (as the ATPase A subunit) and ABCI12 (as the membrane-intrinsic, energy coupling T subunit) in the chloroplast IE represent the energy-coupling module of a novel, prokaryotic type ECF-transporter complex.

## Materials and Methods

### Plant Material and Growth Conditions

All experiments were performed on *Arabidopsis thaliana* ecotype Col-0 (Lehle Seeds, Round Rock, USA). The T-DNA insertion lines SALK_027278 (*abci10-1*), GABI_946_B10 (*abci10-2*), GABI_969_D10 (*abci10-3*), CS16225/EMB2751 (*abci10-4*), and SALK_116866 (*abci11-1*) were purchased from NASC (University of Nottingham), GABI-Kat (Max Planck Institute for Plant Breeding Research), and ABRC (Ohio State University). Please note that *abci11-1* under the name *nap14-1* has been characterized previously by Shimoni-Shor et al. (2010). Before sowing, seeds were surface-sterilized and vernalized at 4°C to synchronize germination.

Plants were grown in ½ Murashige and Skoog (MS) plates containing 0.8% agar and 1-1.5% sucrose in a controlled environment with a 16 h light (100 µmol/m^2^·s)/8 h dark cycle at 22°C/18°C. Due to the sterility of the homozygous knockout lines *abci10-1*, *abci10-3*, *abci10-4*, and *abci11-1*, seeds of heterozygous mutant plants were sown to obtain homozygous mutant progeny. In case grown for more than 3 weeks, only homozygous *abci10* and *abci11* plantlets were transferred to new ½ MS plates. To observe developing seeds, siliques were gently opened by fine forceps at 9–10 days after flowering. To examine growth under excess manganese, homozygous *abci10-1*,* abci10-4*, and *abci11-1* as well as wild-type plants were germinated for 1–2 weeks on control ½ MS plates and subsequently transferred to ½ MS plates supplemented with 0, 0.5, 0.75, or 1.0 mM MnSO_4_. Growth phenotypes were documented 2 weeks after transfer to Mn-supplemented media. Dry weight of *abci10-1*, *abci10-3*, and *abci11-1* homozygous knockout mutants, the corresponding segregated wild-type lines, and Col-0 was determined from 3-week-old seedlings grown on ½ MS media, supplemented with 0, 5, 20, 100, 300, and 1000 µM Fe. Prior to freeze-drying for 4–8 h, 2–15 individual plantlets were sampled per data point. Dry weight was determined by a special accuracy weighing machine.

### GFP, RFP, and YFP Fusion Proteins

To yield the ABCI10-GFP and ABCI11-GFP constructs (lab of JP/YL), the coding sequences of At-ABCI10 and At-ABCI11 without stop codons were ligated into the 326sGFP vector (Clonetech) using the XbaI site. For ABCI11-mRFP and ABCI12-mRFP constructs, the coding sequences of At-ABCI11 and At-ABCI12 without stop codons were ligated into the 326mRFP (Clonetech) vector using the XbaI site. For PIC1-mRFP, the coding sequence of At-PIC1 without stop codon was ligated into the 326mRFP vector (Clonetech) with the BamHI site. The PGL35-YFP construct was a kind gift from Felix Kessler (Vidi et al., 2006).

In a second approach by the group of KP, C-terminal fusions of GFP and YFP to the preproteins of ABCI10, ABCI11, and ABCI12 were constructed by subcloning PCR-amplified cDNA into the pENTR/D/TOPO and further into the p2GWF7(GFP) and pB7YWG2(YFP) plasmid vectors (Karimi et al., 2002) using the Gateway cloning system (Invitrogen). PIC1/pK7FWG2 (Duy et al., 2007b) and FSD1/pPOL (Chang et al., 2014) were used as controls.

### Protoplast Isolation and Transient Expression

Wild-type *Arabidopsis* plants were grown on soil pots and mesophyll protoplasts were isolated as described (Duy et al., 2007b) from rosette leaves by enzymatic digestion using an enzyme solution [400 mM mannitol, 20 mM KCl, 20 mM MES-KOH pH 5.7, 10 mM CaCl_2_, 0.25% w/v Macerozyme R-10 (Yakult), 1% w/v Cellulase R-10 (Yakult), 0.1% w/v bovine serum albumin]. Healthy mesophyll protoplasts were transformed by the polyethylene glycol method (Abel and Theologis, 1998; Duy et al., 2007b), and fluorescent signals were observed using the respective microscopes. To isolate protoplasts from *abci10-1* and *abci11-1* homozygous mutants, plants were grown on ½ MS plates for 3–4 weeks, and whole plants were used for enzymatic digestion using the enzyme solution. Overnight incubation of the mutant plants in the enzyme solution improved protoplast isolation.

### Microscopy

Confocal images in the lab of JP/YL were observed using an Olympus FV1000 confocal laser scanning microscope (Olympus) with spectral settings of excitation at 488 nm and emission at 500–530 nm for GFP, ex 543 nm/em 575–630 nm for RFP, and ex 515 nm/em 520–560 nm for YFP. Fluorescent images were observed using a Zeiss Axioskop2 microscope (Zeiss) with spectral settings of excitation at 455–495 nm and emission at 505–555 nm for GFP, ex 540–552 nm/em 575–640 nm for RFP, and ex 546/12 nm/em 575–640 nm for chlorophyll autofluorescence.

Images in the group of KP were recorded with a confocal laser scanning microscope (Leica, TCS SP5). Here, protoplasts were examined using the 63x1.3 glycerine-immersion objective with excitation using the argon laser (ex 488 nm for GFP, ex 524 nm for YFP). The emitted light of GFP and YFP was detected at 509 nm and 527 nm, respectively. Chlorophyll auto-fluorescence was monitored at 497–524 nm. When appropriate, the bright field images of samples were imaged with the transmitted light photomultiplier. All images of LVvV, RS were processed with Leica LA SAF Lite (Leica).

Ultrastructural analysis by transmission electron microscopy was conducted as described (Duy et al., 2007b).

### Measurement of Ion and Chlorophyll Contents

Homozygous *abci10* and *abci11* mutant plants were grown in ½ MS plates for 34 days, and shoots or whole tissues were collected separately for ICP-MS measurements. Wild-type plants were grown for 15 or 16 days. Plant tissues were rinsed twice with 2 mM K^+^- phosphate buffer (pH 5.7) and once with ice-cold water. Samples were digested with 11 N HNO_3_ at 100°C for 6 h to 1 day. After samples were completely digested, they were diluted with distilled water, and ion contents were analyzed using an ICP-MS spectrometer (ELAN DRC-e; Perkin-Elmer). Ni and Mo contents as well as chlorophyll were determined in 20-day-old seedlings as described (Duy et al., 2007b; Duy et al., 2011).

### Transcript Level Profiling

Quantitative real-time RT-PCR was performed as described previously (Duy et al., 2007b) using a LightCycler (Roche). All signals were normalized to the signal of actin cDNA fragments from *Actin* 2 and 8 (At3g18780 and At1g49240).

### Antiserum Production

To raise antisera against At-ABCI10 and At-ABCI11, cDNA was PCR-amplified on the respective SALK pUNI clones U62184 and U51365 (Yamada et al., 2003). The resulting mature versions of At-ABCI10 and At-ABCI11 were subcloned into the pET21d (Novagen) plasmid vector and used for overexpression after transforming *E. coli* BL21(DE3) cells (Novagen). Rapidly growing cells with an OD_600_ of 0.6 were induced with 0.6 mM isopropyl1-thio-b-D-galactopyranoside (IPTG) for 3 h at 37°C. Afterwards, pelleted cells (4°C, 6,000g, 15 min) were resuspended in cell lysis solution (50 mM Tris-HCl, pH 8.0, 25% [w/v] sucrose, 1 mM EDTA, 100 mg/ml DNase) and sonicated three times for 30 s. Inclusion bodies were collected by centrifugation at 4°C and 20,000g for 30 min. The resulting pellet was resuspended in buffer A (50 mM NaPP, pH 8.0, 100 mM NaCl, 2 mM β-mercaptoethanol, and 8 M urea), and remaining insoluble material was separated by centrifugation. The major fraction of overexpressed recombinant At-ABCI10 and At-ABCI11 protein was present in the urea-soluble supernatant. Both proteins were purified *via* their C-terminal polyhistidine tags using Ni-NTA-Sepharose as described (Harsman et al., 2016) and eluted in buffer A including increasing imidazole concentrations (100–500 mM). After addition of 1% [w/v] SDS, the purified recombinant mature At-ABCI10 and At-ABCI11 proteins were used as antigenes to raise antibodies in rabbit (Pineda Antibody Service). Antisera for marker proteins were produced as described previously (Küchler et al., 2002; Duy et al., 2007b; Philippar et al., 2007).

### Immunoblot Analysis

For immunoblot analysis, pea and *Arabidopsis* chloroplasts were isolated and subfractionated as described (Duy et al., 2007b; Li et al., 2015). Total proteins from seedling tissue grinded in liquid nitrogen were extracted for 30 min on ice in buffer (50 mM Tris-HCl, pH 8.0; 50 mM EDTA; 2% LDS; 10 mM DTT; 100 mM PMSF). Cell debris was pelleted for 15 min at 14,000g, 4°C.

For protein extraction and solubilization, pea IE membrane vesicles were pelleted by centrifugation at 256,000g, 4°C for 10 min and resuspended in either 1 M NaCl, 0.1 M Na_2_CO_3_ (pH 11.3), 6 M urea, or 1% Triton X-100, followed by incubation for 20 min on ice or for urea extraction at RT. Afterwards, IE membranes corresponding to 20 μg protein for each assay were separated into membranes and solubilized proteins by centrifugation at 100,000g, 4°C for 10 min.

All protein fractions were separated by SDS-PAGE and transferred to Immobilon-P PVDF membrane (Millipore) for immunoblot analysis. Polyclonal antisera against recombinant At-ABCI10 and At-ABCI11 proteins were used in 1:500 and 1:2,000 dilution (0.1 M Tris-HCl pH 7.5, 0.15 M NaCl, 0.2% Tween 20). The antisera against At-PIC1, Tic62, LHC, and pSSU were diluted 1:1,000–1:5,000, and serum against OEP16.1 was used in 1:500 dilution. Secondary, alkaline phosphatase coupled antibodies (Sigma-Aldrich) were diluted 1:5,000. Nonspecific signals were blocked by 1% or 3% skim milk powder and 0.03% or 0.1% BSA. Blots were stained using the alkaline phosphatase reaction in the presence of nitroblue tetrazolium and bromochloroindolyl phosphate as substrate.

### Co-Immunoprecipitation

Coupling of protein-A-sepharose to the antibodies against At-ABCI10 and At-ABCI11 or the respective pre-immune sera was done in advance. Therefore, for each assay, 40 µl of protein-A-sepharose was incubated with 10 µl of the antiserum in 0.2 M triethanolamine (pH 8.2) at room temperature for 1 h. Following a washing step with triethanolamine, coupling was achieved using 20 mM dimethyl pimelimidate and incubation at RT for 1 h. Unspecific binding was reduced by incubation of the sepharose-antibody suspension with triethanolamine for 1 h. Following three washing steps with IP buffer (50 mM Tris/HCl, pH 7.5, 150 mM NaCl), the antibody-coupled sepharose was kept at 4°C for 20 h.

Isolated and purified pea IE membranes (corresponding to 70 µg protein) were solubilized with 0.5% dodecylmaltoside in IP buffer on ice for 1 h by repeated pipetting (every 10 min). After centrifugation at 100,000g for 10 min, 4°C, the protein-containing supernatant was diluted 1:10 in IP buffer. Subsequently, the solubilized and diluted IE proteins were added to the antibody-coupled protein-A-sepharose and incubated at room-temperature for 2 h. Sepharose beads were washed three times in IP buffer with 0.05% dodecylmaltoside and once with IP buffer without detergent. Elution was performed using SDS PAGE loading buffer without ß-mercaptoethanol, and separation from the sepharose beads was achieved using a Micro Bio-Spin Chromatography Column (BioRad). Load, flow-through, washes, and elution fractions were analyzed by SDS-PAGE and immunoblotting.

## Results

**Figure 1:**
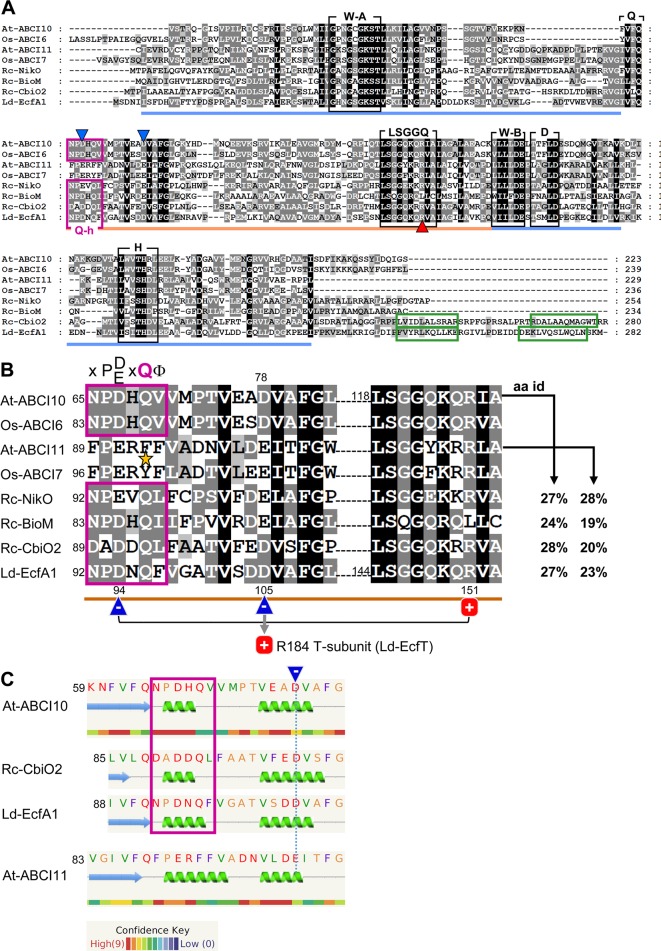
ABCI10 and ABCI11 proteins. **(A)** Amino acid sequences of mature *Arabidopsis* At-ABCI10 (Q8H1R4), At-ABCI11 (Q8LEF6), rice Os-ABCI6 (Q5ZD09), Os-ABCI7 (Q2R434), and the bacterial ECF transporter A subunits NikO (D5AQY6), BioM (D5ARH0), CbiO2 (O68106) from *Rhodobacter capsulatus* and EcfA1 (Q1GBJ0) from *Lactobacillus delbrueckii*. UniProtKB accession numbers are given in brackets (UniProt Consortium, 2018), and chloroplast targeting peptides were predicted by ChloroP (Emanuelsson et al., 1999). Please note that in rice, the protein Os-ABCI7 (Q2R434) was named Os-ABCI8 by Zeng et al. (2017). The conserved motifs Walker A, Walker B, Q-loop, D-loop, and H-loop (black boxes) form the nucleotide binding sites in the RecA domain (blue line). The ABC-transporter ATPase specific helical subdomain (orange line) contains the signature motif LSGGQ. The Q-helix (purple box) and the two α-helices in the C-terminal domain of CbiO2 and EcfA1 (green boxes) are indicated. Conserved negative acidic residues are highlighted by blue, and the conserved positively charged arginines are highlighted by red triangles. All motifs and domains are depicted according to the crystal structures of Rc-CbiO2 and Ld-EcfA1 in the CbiMQO and FolT2 complexes (pdb entries 5X3X and 5D7T). **(B)** Highlighted Q-helix (purple box) and helical subdomain region from alignment in **(A)**. In ECF transporter A subunits, the Q-helix with the specific motif X-P-D/E-X-Q-Φ (X is any, Φ a hydrophobic amino acid) directly follows the Q-loop that connects the RecA and helical subdomain [see **(A)**]. The invariant glutamine (Q) residue of the Q-helix is absent in ABCI11 proteins (asterisk). According to the crystal structure of FolT2 (Swier et al., 2016; Rempel et al., 2019), the conserved acidic and negatively charged residue (blue triangle) in the Q-helix of EcfA1 (D_94_) forms an intramolecular interaction with the conserved positive arginine (R_151_) in the LSGGQ motif (red square). Further, the invariant acidic residue in the first helix of the helical subdomain (D_105_ of LcfA1) is positioned at the surface of the negatively charged groove of the EcfA1A2 dimer and binds to the conserved arginine (R_184_) in the coupling helix 2 of the membrane-intrinsic EcfT subunit (compare **Supplementary Figure 10**). The amino acid identities (aa id) of the full mature At-ABCI10 and At-ABCI11 proteins to Rc-NikO, Rc-BioM, Rc-CbiO2, and Ld-EcfA1 are indicated. **(C)** Secondary structure and disorder prediction by Phyre2 (Kelley et al., 2015) reveals that the Q-helix predicted for At-ABCI10 most likely forms a short helical turn specific for ECF transporter ATPase subunits like CbiO2 and EcfA1. In contrast, substitution of the conserved polar glutamine (Q) by a hydrophobic phenylalanine (F) in At-ABCI11 leads to a less confident prediction of a longer helix. In consequence, the acidic glutamate in the first α-helix of the helical subdomain of At-ABCI11 (dotted blue line) might not be positioned for proper interaction with residues of a membrane-intrinsic T-subunit.

Our database and literature search for prokaryotic-like ABC-transporter subunits, which might contribute to chloroplast metal transport in *Arabidopis* identified At-ABCI10 (At4g33460), At-ABCI11 (At5g14100), and At-ABCI12 (At3g21580) as promising candidates. All three proteins are predicted to be targeted to chloroplasts and were grouped into the prokaryotic-type CBY/Y179 subfamily of *Arabidopsis* and rice ABC transport systems (Garcia et al., 2004; Verrier et al., 2008), annotated with a potential function in metal transport. ABCI10 and ABCI11—also known as NAP13 and NAP14 for non-intrinsic ABC protein 13 and 14, respectively—are soluble NBD ATPase-subunits. Since the two proteins are encoded by separate genes and do not include a TMD-permease subunit, which is common for eukaryotic full- or half-size ABC transporters, ABCI10 and ABCI11 belong to group I of prokaryotic-type, multisubunit ABC-transporters in plants (Verrier et al., 2008). At-ABCI11/NAP14 has previously been reported to localize to the chloroplast stroma and has already been implicated with a function in chloroplast Fe-homeostasis (Shimoni-Shor et al., 2010). Similar results were obtained for the orthologous protein Os-ABCI7 in rice (*Oryza sativa*; Zeng et al., 2017). Please note that Zeng and co-authors (2017) unfortunately named this protein Os-ABCI8, whereas the consortium-annotated name in Verrier et al. (2008) is Os-ABCI7 for the NBD-subunit orthologous to At-ABCI11/NAP14 (compare **Figure 1A**). Os-ABCI8 instead is the consortium and database name for the rice transmembrane-protein relative to At-ABCI12. ABCI12 in *Arabidopsis* also is encoded by a separate gene, is predicted to contain five transmembrane α-helices, and most likely represents the membrane-intrinsic T subunit of a non-classical, prokaryotic-type ECF ABC-transporter complex that was identified by *in silico* analyses and named “plant T protein” (Eitinger et al., 2011). In general, plant ABCI10, 11, and 12 polypeptides are nucleus encoded and contain predicted N-terminal chloroplast targeting peptides (Aramemnon database; Schwacke et al., 2003). In the AT_CHLORO proteome database, all three proteins are assigned to the chloroplast envelope (Ferro et al., 2010). However, only for ABCI10, peptides have been experimentally detected in purified “mixed” envelopes from *Arabidopsis* (Froehlich et al., 2003), envelope preparations from maize (*Zea mays;* Bräutigam et al., 2008), and IE membranes from pea (*Pisum sativum;* Gutierrez-Carbonell et al., 2014).

### ABCI10 and ABCI11 Are Prokaryotic-Type NBD-Domain ATPase Subunits

In *Arabidopsis*, the mature proteins ABCI10 and ABCI11 are predicted to be 223aa and 229aa long with a molecular mass of 24.4 kDa and 25.3 kDa, respectively. Both contain the classical ATPase motifs Walker A, Walker B, Q-, D-, and H-loop, which form the nucleotide-binding site (Gaudet and Wiley 2001) as well as the ABC transporter signature motif LSGGQ in the helical subdomain specific for NBD subunits of ABC transporters (Davidson et al., 2008; Wilkens, 2015) (**Figure 1A**). Furthermore, an “ABC_cobalt_CbiO_domain 1” [conserved protein domain family (CDD) cd03225] is annotated to span the entire sequence of the mature At-ABCI10 and At-ABCI11 proteins (GenPept entries NP_195072, NP_196914). CbiO in bacteria has been shown to be the ATPase subunit A in group I ECF-transporters named CbiMNQO for import of cobalt ions (Rodionov et al., 2006; Rodionov et al., 2009), which has recently been crystallized from *R. capsulatus *as CbiMQO core (Bao et al., 2017). Since At-ABCI10 and At-ABCI11 represent separately encoded NBD subunits of ABC transporter complexes, their prokaryotic origin seems obvious (Verrier et al., 2008; Theodoulou and Kerr, 2015). Both proteins are conserved in the “green lineage” with relatives in dicots, monocots, mosses, green microalgae, and cyanobacteria (**Supplementary Figures 1**, **2**).

Although ABCI10 and ABCI11 include all domains of ABC transporter ATPases, their amino acid sequences and predicted secondary structures show some differences. While At-ABCI10 and the identified relatives from plants and cyanobacteria contain the so-called Q-helix motif, described to be specific for ATPase A components of bacterial ECF transporters (Karpowich and Wang, 2013; Rempel et al., 2019), this X-P-D/E-X-Q-Φ consensus sequence is not conserved in ABCI11 proteins (**Figure 1**, **Supplementary Figures 1**, **2**). In comparison to Q-helix motifs of ECF A subunits from *R. capsulatus* (NikO, BioM, CbiO2) and from *L. delbrueckii* (EcfA1), ABCI10 from *Arabidopsis* and ABCI6 from rice have the amino acid stretch N-P-D-H-Q-V, including the conserved proline (P), acidic aspartate (D), and glutamine (Q) residues (**Figure 1A,B**). Thus, a short α-helical turn is predicted for the Q-helix of ABCI10, very similar to that of the crystallized CbiO2 and EcfA1 proteins (**Figure 1C**). In general, the Q-helix motif directly follows the Q-loop region and thereby is placed at the beginning of the helical subdomain of ECF transporter ATPases. The detailed structures of CbiO2 and EcfA1/A2 in the complexes CbiMQO (Co-uptake) and ECF-FolT (folate transport) (Swier et al., 2016; Bao et al., 2017) reveal that the Q-helix is essential for interaction of a conserved aspartate residue in the helical subdomain of A subunits (D_102_ of CbiO2, D_105_ of EcfA1) with a conserved arginine motif in the coupling helices of subunit T (**Figure 1B**). Furthermore, another invariant acidic residue in the Q-helix itself can form an intramolecular interaction with a conserved arginine in the LSGGQ motif of each A subunit. In contrast to ABCI10-family proteins, the full Q-helix motif is not present in At-ABCI11 and its plant or cyanobacterial relatives (**Figure 1**, **Supplementary Figure 2**). In At-ABCI11 and Os-ABCI7, the invariant polar glutamine (Q) residue is replaced by hydrophobic phenylalanine and tyrosine residues, respectively (**Figure 1B**). In consequence, the predicted α-helix in the amino acid stretch corresponding to the Q-helix is longer and less confident for At-ABCI11 (**Figure 1C**). Thus, the correct and ECF-specific formation of the groove in ABCI11 ATPase subunits for specific interaction with membrane-intrinsic T-components of an ECF transporter might not be possible.

Therefore, we propose that At-ABCI10 most likely represents an ATPase A subunit of a prokaryotic-type ECF transporter. ATPases of all prokaryotic group II ECF transporters (like EcfA1 from FolT) and of some group I proteins (like CbiO2 from CbiMNQO, compare **Figure 1A**) are characterized by an additional C-terminal helical extension that is proposed to act in dimer formation in the absence of ATP and to have a regulatory function (Rodionov et al., 2009; Rempel et al., 2019). Since this C-terminus is absent in ABCI10, the protein most likely groups to type-I ECF transporters like NikO from NikMNQO or BioM from BioMNY (**Figure 1A**). In contrast to ABCI10, At-ABCI11/NAP14 might correspond to an ATPase NBD-domain subunit of a canonical prokaryotic-type ABC transporter similar to TGD3/ABCI13 in the TGD complex for lipid import into chloroplasts (Lu et al., 2007) or to other organelle intrinsic NAP proteins. This hypothesis is further supported by the finding that *in silico* structural modeling (Phyre2; Kelley et al., 2015) for At-ABCI10 gives the most likely hit to the ECF transporter A subunit CbiO2 from *R. capsulatus* (PDB database entry 5X3X; 100% confidence, 37% aa identity). At-ABCI11 instead shows the most similar structure (100% confidence, 33% identity) to the NBD subunit of a hypothetical prokaryotic maltose/maltodextrin ABC transporter (PDB database entry 2IT1). When comparing all single ATPase NBD-subunits from the ABCI family of *Arabiodpsis* ABC transporters (Verrier et al., 2008), only At-ABCI10 has the full characteristic Q-helix consensus motif, indicating that this protein might be the only prokaryotic-type ECF ATPase in the dicot model plant (**Supplementary Figure 3**).

### ABCI10 and ABCI11 Attach to Chloroplast-Intrinsic Membranes

**Figure 2:**
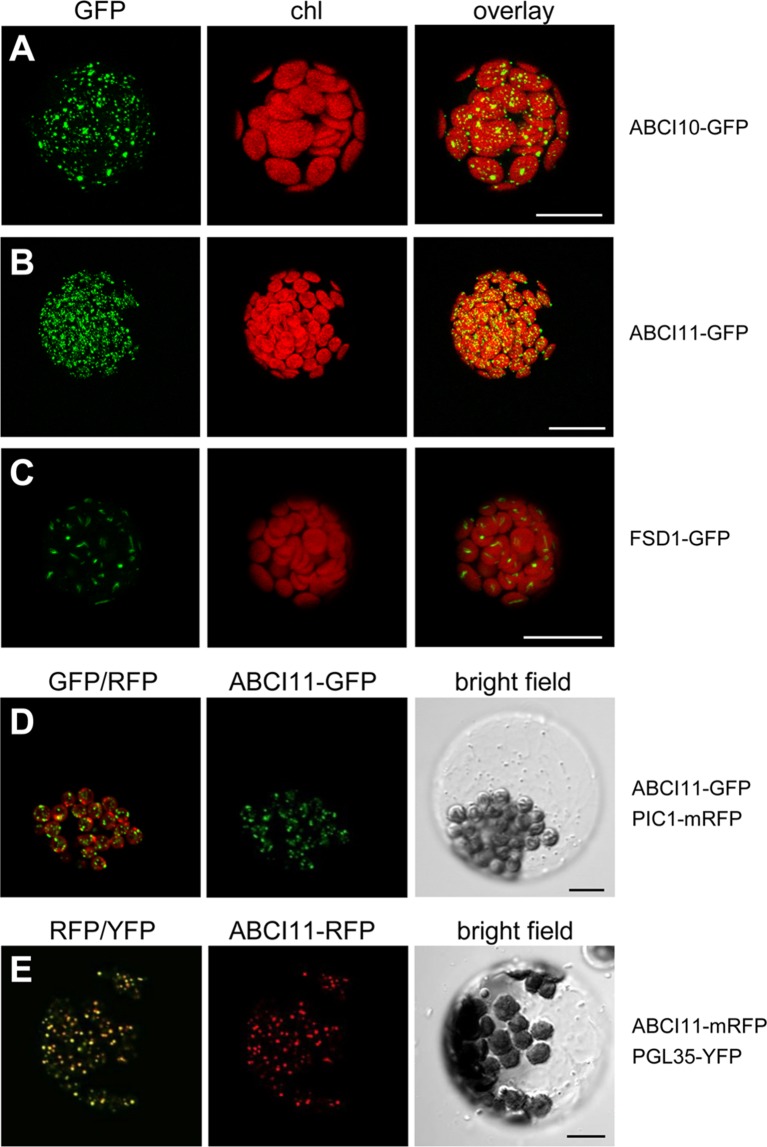
At-ABCI10 and At-ABCI11 are targeted to chloroplasts. *In vivo* GFP-targeting of At-ABCI10 and At-ABCI11. *Arabidopsis* leaf protoplasts were transiently transformed with constructs for At-ABCI10-GFP **(A)**, At-ABCI11-GFP **(B)**, FSD1-GFP [chloroplast stroma marker; (Chang et al., 2014)] **(C)**, as well as At-ABCI11-GFP co-expressed with PIC1-mRFP **(D)** and At-ABCI11-mRFP co-expressed with PGL35-YFP **(E)**. Images in **(A–C)** show GFP-signals (left), chlorophyll fluorescence (middle), as well as an overlay of both (right). Images in **(D,E)** display an overlay of the respective GFP/RFP and RFP/YFP fluorescence (left), signals of At-ABCI11 constructs (middle) as well as a bright field image of the protoplast. Scale bars = 10 µm.

To verify the subcellular localization of At-ABCI10 and At-ABCI11, we performed *in vivo* GFP-targeting assays by transiently transforming *Arabidopsis* leaf mesophyll protoplasts with the ABCI proteins tagged with fluorescent proteins at the C-terminal end (**Figure 2**). Protoplasts transformed with ABCI10-GFP or ABCI11-GFP both exhibited fluorescence signals only in chloroplasts as suggested by sequence prediction (**Figures 2A, B**). However, signal patterns of GFP tagged to ABCI10 and ABCI11 were slightly dissimilar and clearly different from the stroma-targeted protein control FSD1 (Fe-superoxide dismutase 1; Chang et al., 2014; Kliebenstein et al., 1998). In each chloroplast, FSD1-GFP proteins represented a single signal inside the stroma that was surrounded by the bowl-shaped thylakoid membrane systems (**Figure 2C**). In contrast, the multiple punctuate stains of ABCI10-GFP seemed to be more around the chloroplasts periphery, suggesting its localization at the envelope region (**Figure 2A**). Distribution of ABCI11-GFP fluorescence was more uniform as shown by numerous dots all-over each chloroplast (**Figure 2B**). For fluorescence signals by non-chloroplast targeted cytosolic GFP, please see Chang et al. (2014). To examine these unexpected GFP-signals of ABCI11 in more detail, we co-expressed ABCI11 tagged proteins with chloroplast-intrinsic marker proteins PIC1-RFP (**Figure 2D**) or PGL35-YFP (Vidi et al., 2006; **Figure 2E**). The Fe-permease PIC1 is localized in the IE of chloroplasts (Duy et al., 2007b), while the plastoglobulin PGL35 is mostly targeted to plastoglobuli, which are plastid-intrinsic lipoprotein particles surrounded by monolayer lipid membranes (Shanmugabalaji et al., 2013). While signals for ABCI11-GFP did not show any overlap with those for PIC1-RFP at the envelopes surrounding the chloroplasts (**Figure 2D**), the superimposition of ABCI11-RFP and PGL35-YFP fluorescence was almost complete (**Figure 2E**), suggesting attachment to ABCI11 to plastoglobuli (van Wijk and Kessler, 2017).

**Figure 3:**
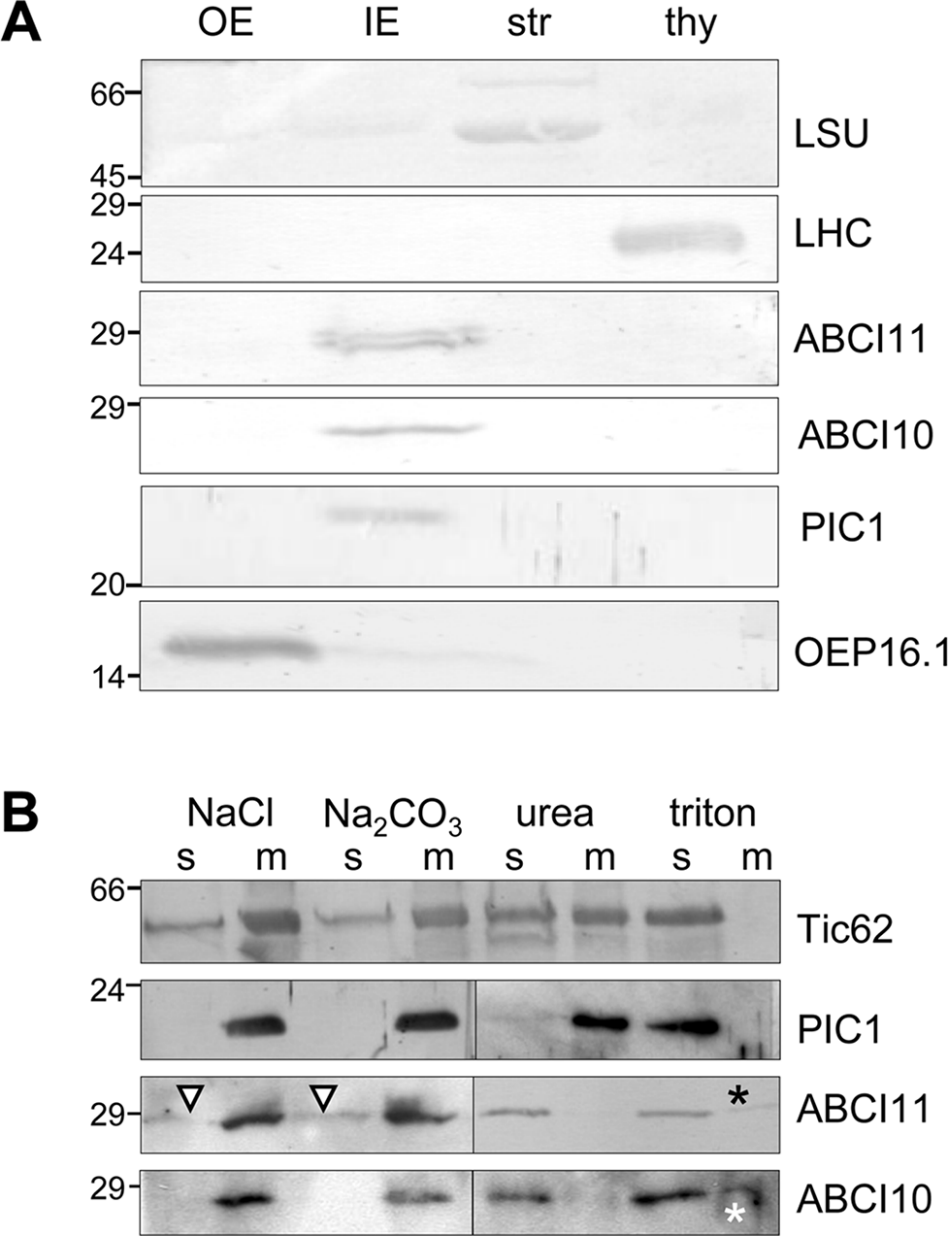
ABCI10 and ABCI11 attach to chloroplast IE membranes. Immunoblot analysis of ABCI10 and ABCI11 localization in chloroplast subfractions. Numbers indicate molecular mass of proteins in kDa. **(A)** Intact isolated chloroplasts from pea (*Pisum sativum*) were fractionated into outer (OE) and inner (IE) envelope membranes, stroma (str) and thylakoids (thy). Equal protein amounts (10 µg) were separated by SDS-PAGE and were subjected to immunoblot analysis using antisera against the mature proteins At-ABCI10 and At-ABCI11, respectively. Antisera against the marker proteins LSU (large subunit rubisco, stroma), LHC (light harvesting complex, thylakoids), OEP16.1 (outer envelope protein of 16 kDa, OE), and PIC1 (Fe-uptake, IE) were used as controls. **(B)** After treatment with either high ionic strength (1M NaCl, pH 8), high pH buffer (Na_2_CO_3_, pH 11.3), denaturing agent (6 M urea) or detergent (1% Triton X-100), pea IE membranes (20 µg protein for each assay) were fractionated into insoluble, membrane-intrinsic (m) and soluble proteins (s) by ultracentrifugation. Subsequently, proteins were separated by SDS-PAGE and subjected to immunoblot analysis using antisera against At-ABCI10 and At-ABCI11. Antisera against the marker proteins Tic62 (loosely attached to IE membranes) and PIC1 (IE membrane-intrinsic, hydrophobic permease) were used as controls. Triangles and asterisks indicate trace amounts of ABCI11 in soluble fractions after high salt, high pH treatment and residual signals of ABCI10, ABCI11 in membrane fractions after detergent solubilization, respectively.

To confirm the results of *in vivo* GFP-targeting assays, which sometimes could be misleading due to artificial overexpression, we further tested for subcellular localization of ABCI10 and ABCI11 proteins by immunoblot analysis using isolated and sub-fractionated chloroplasts (**Figure 3**). Therefore, we generated antisera against the purified recombinant mature proteins At-ABCI10 and At-ABCI11 (**Supplementary Figure 4**). Immunoblot analysis using chloroplast membranes from pea (*Pisum sativum*; **Figure 3A**) showed that ABCI11 runs at a molecular weight of about 28–29 kDa, well in line with the size of the purified, recombinant mature At-ABCI11 protein (**Supplementary Figure 4**). In the pea IE fraction, the antiserum directed against *Arabidopsis* ABCI11 stains a double band that might be due to unspecific interaction, possibly with another chloroplast NBD-protein from pea or to alternative processing of the mature polypeptide. Instead, α-At-ABCI10 in pea IE stains a single band at 27 kDa (**Figure 3A**), nicely reflecting the size of the recombinant protein (**Supplementary Figure 4**) and the fact that the mature ABCI10 is about 1 kDa smaller than ABCI11. In addition, we tested both antisera on *Arabidopsis* chloroplast and tissue preparations from wild type and *abci10*, *abci11* knockout mutants, where we could assign signals to endogenous At-ABCI10 and At-ABCI11 proteins (**Supplementary Figure 5**). Surprisingly, the immunoblot analyses showed ABCI10 and ABCI11 only in chloroplast IE membrane fractions and not in the stroma, as would be expected for soluble ATPase proteins without any transmembrane domains (**Figure 3**). The purity of the pea IE membranes was confirmed with controls against the stroma marker protein LSU, the thylakoid marker protein LHC, the OE protein OEP16.1, and the IE-intrinsic PIC1 (**Figure 3A**).

To follow the observed unusual attachment of soluble ABCI10 and ABCI11 proteins to the IE membrane of chloroplasts in more detail, we treated purified pea IE vesicles with high ionic strength and high pH buffers as well as denaturing and detergent agents (**Figure 3B**). Subsequent separation of membranes by centrifugation, followed by immunoblot analysis can reveal if proteins are still attached to/intrinsic to membranes or upon treatment are dissolved into the soluble fraction. In our assays, neither high salt nor high pH could detach both ABCI10 and ABCI11 from the membrane pellet (**Figure 3B**, left panel). Since ABCI10 and ABCI11 do not contain any membrane-spanning, hydrophobic domains, this indicates that most likely both proteins attach to the IE membrane *via* strong interactions with a membrane-embedded anchor protein. For ABCI11, this interaction might be slightly weaker and/or different, because some protein traces were washed from the membrane to the supernatant by salt and high pH treatment (**Figure 3B**, triangle). Denaturation of proteins and membranes by urea as well as membrane solubilization by the detergent Triton X-100 successfully moved ABCI10 and ABCI11 proteins to the soluble supernatant (**Figure 3B**, right panel). Thus, upon urea treatment, the potential anchor proteins and interaction sites for ABCI proteins would be denatured and therefore release ABCI10 and ABCI11 from the membranes. Some residual amounts of proteins in the membrane fraction after detergent usage most likely are due to incomplete solubilization of membranes and anchor proteins. As control we used the antiserum for PIC1, which contains four predicted hydrophobic, membrane-spanning α-helical domains (Duy et al., 2007b; Duy et al., 2011). PIC1—as expected for an IE transmembrane protein—was detected in the soluble fraction only after membrane disruption by Triton X-100. Instead, Tic62, which attaches more loosely to the inner leaflet of the IE membrane (Küchler et al., 2002; Stengel et al., 2008), was already partly solubilized by high salt, high pH and urea (**Figure 3B**).

In summary, our results on the subcellular and suborganellar localization of ABCI10 and ABCI11 point to their strong attachment to chloroplast membranes possibly *via* interaction with potential membrane-intrinsic subunits of prokaryotic-type ABC transporter complexes. ABCI10 is most likely anchored to the IE membrane, while ABCI11 might be attached to the lipid monolayer of plastoglobuli that might co-purify with IE membranes upon isolation.

### The Loss of ABCI10 as well as of ABCI11 Impairs Plant Growth and Chloroplast Biogenesis

**Figure 4:**
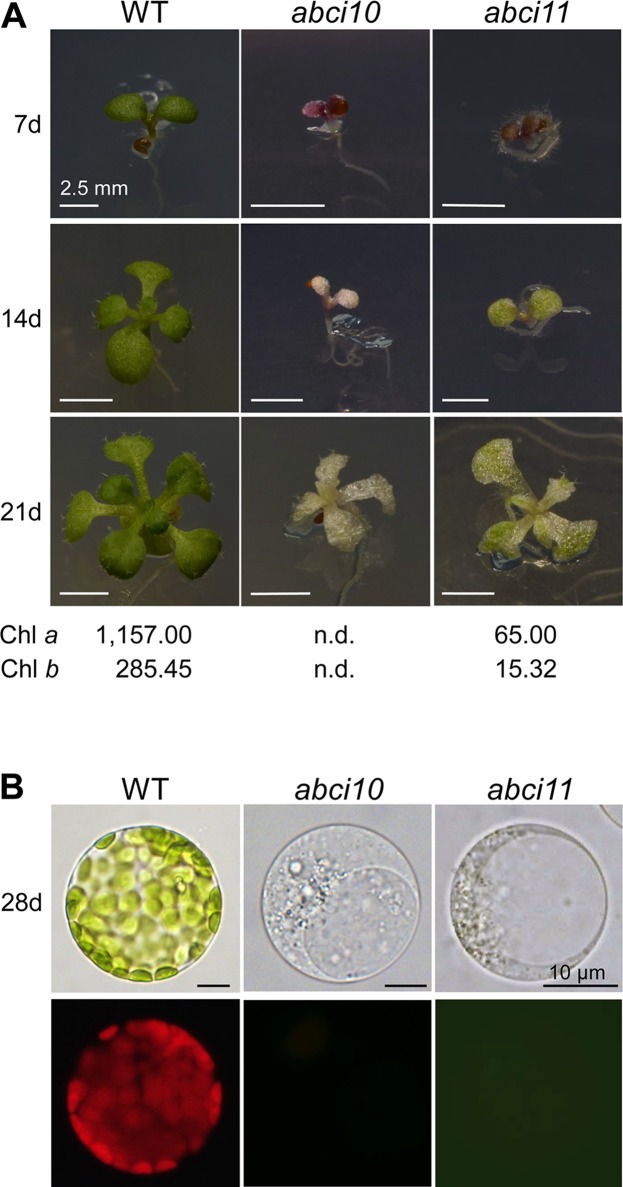
ABCI10 and ABCI11 loss-of-function mutants are dwarfed albino plants. **(A)** Seedlings of Col-0 wild type, *abci10-1*, and *abci11-1* knockout mutants grown for 7, 14, and 21 days on agar medium supplemented with sucrose. Scale bars = 2.5 mm. Chlorophyll *a* and chlorophyll *b* content was measured in 21-day-old plantlets. Mean values (ng chlorophyll/mg freshweight) from two independent extractions on each time 9 (Col-0), 16 (*abci10*), and 26 (*acbi11*) pooled individuals are shown. n.d., not detectable. **(B)** Protoplasts isolated from 28-day-old seedlings depicted in **(A)**. The upper panel shows bright field images, which document absence of mature chloroplasts in *abci10-1* and *abci11-1* mutants. The lower panel depicts chlorophyll autofluorescence of the same protoplasts. Please note that plastid structures in protoplasts of *abci11-1* appear to be slightly greenish. Scale bars = 10 µm.

To follow the role of ABCI10 and ABCI11 *in planta*, we characterized the mutant lines *abci10-1*, *abci10-2*, *abci10-3*, *abci10-4*, and *abci11-1* in *Arabidopsis* (**Supplementary Figures 5**, **6**, **7**). Please note that under the name *nap14-1*, the line *abci11-1* was already described in detail by Shimoni-Shor et al. (2010). The generation of specific antisera, however, now enabled us to probe for protein levels (**Supplementary Figure 5D**). Both, the loss of either At-ABCI10 or At-ABCI11 proteins led to impaired segregation of homozygous mutant alleles, most likely due to partial embryo and/or seed lethality (**Supplementary Figure 5E, F**). For *abci10*, the observed embryo lethality is well in line with the annotation of the protein as “embryo defective 2751” (Universal Protein resource UniProtKB - Q8H1R4; UniProt Consortium, 2018). Homozygous plantlets of knockout lines, namely, *abci10-1*,* abci10-3*, *abci10-4*, and *abci11-1*, did not survive on soil, but had to be grown on sucrose-supplemented media and mutant lines had to be propagated in the heterozygous state. Knockout mutant plantlets for *At-ABCI10* or *At-ABCI11* were characterized by an extremely dwarf and chlorophyll-less phenotype (**Figure 4A** and **Supplementary Figures 6D**, **7A, B**). The line *abci10-2* with a T-DNA insertion in the 3’ untranslated region of *At-ABCI10* (**Supplementary Figure 5A**) and therefore not representing a loss-of-function mutant, however, did not show a chlorotic appearance in the homozygous state (**Supplementary Figure 7A**). Leaf structures of *abci10*, *abci11* knockouts were deformed, and mesophyll cells of seedlings were smaller than wild type and did not contain fully developed chloroplasts with chlorophyll (**Figure 4B**). For *ABCI11*, this phenotype has been described previously for *abci11-1/nap14-1* (Shimoni-Shor et al., 2010) and mutation of the corresponding rice ortholog (Zeng et al., 2017). In our phenotype analysis, *abci11-1* showed some trace amounts of green color in seedling leaves as well as in plastid structures of isolated protoplasts (**Figure 4**). By chlorophyll analysis, we here could show that *abci11-1* seedlings still contain residual Chl *a* and Chl *b* (about 18-fold less than Col-0 wild type), whereas the green pigments were below the detection limit in *abci10-1* (**Figure 4A**). Seedling size as well as fresh and dry weight (**Supplementary Figure 7B**) however, did not differ significantly between *abci10* and *abci11* knockout plants. Thus, we can conclude that the albino phenotype of *abci10* knockout lines is more severe than that of *abci11*.

**Figure 5:**
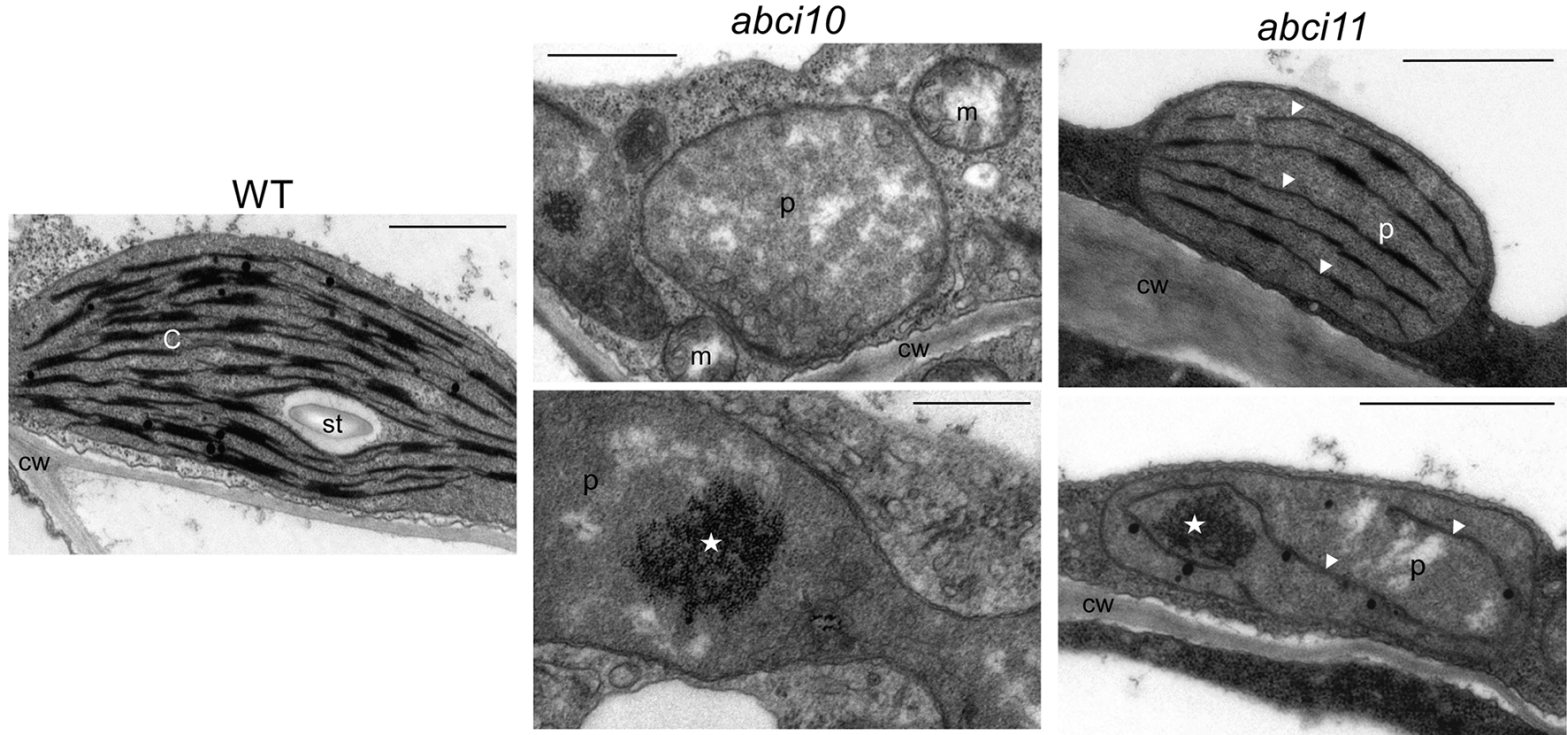
Chloroplast biogenesis is impaired in *abci10-1* and *abci11-1* knockouts.Transmission electron microscopic pictures of plastids from 21-day-old *abci10-1* and *abci11-1* mutant and Col-0 wild-type seedlings. Asterisks indicate electron dense clusters visible in plastids of *abci10-1* and *abci11-1* mutants. Arrowheads highlight prothylakoid-like membranes in *abci11-1*. Scale bars = 1 µm. c, chloroplast; cw, cell wall; m, mitochondrium; p, plastid; st, starch grain.

Ultrastructural analysis of plastids by transmission electron microscopy confirmed the strongly impaired chloroplast biogenesis in *abci10-1* as well as in *abci11-1* mutants (**Figure 5**). Whereas thylakoid membrane systems were completely absent in *abci10-1*, some prothylakoid like structures were detected in *abci11-1* plastids, again pointing to a more severe effect of the loss of At-ABCI10 function. Common to both mutant lines, however, was some electron-dense material that accumulated in the plastid stroma (**Figure 5**).

In summary, the loss of At-ABCI10 as well as of At-ABCI11 function causes a strong dwarf and albino phenotype, and severely affects chloroplast and in particular thylakoid biogenesis, a phenotype that is reminiscent of de-regulated metal homeostasis (Varotto et al., 2002; Ravet et al., 2009; Briat et al., 2010; Shimoni-Shor et al., 2010; Duy et al., 2011). In particular, the completely albino appearance of the three independent *abci10* knockout mutant lines (*abci10-1*, *abci10-3*, *abci10-4*) is very similar to that of loss-of-function mutants of the chloroplast Fe-uptake permease PIC1 (Duy et al., 2007b). Since the chlorotic phenotype is quite pleiotropic, we can, however, not exclude involvement of secondary effects or other metabolic pathways. The difference in the strength of chlorosis of *abci10* and *abci11* confirms the observed potential diverse suborganellar localization and suggests slightly different contribution of ABCI10 and ABCI11 to cellular metabolism.

### ABCI10 and ABCI11 Are Crucial for Metal Homeostasis

**Figure 6:**
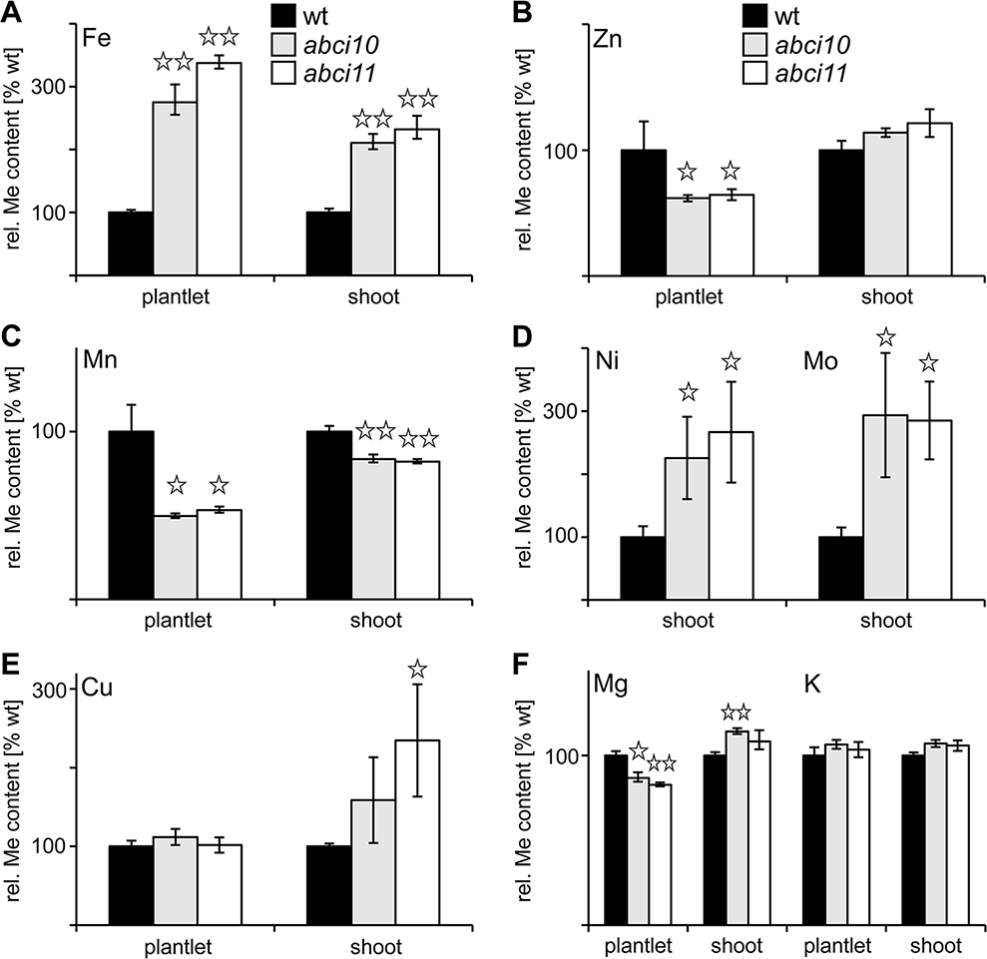
Knockouts of *At-ABCI10* and *At-ABCI11* have a de-regulated metal content. Metal contents for iron **(A)**, zinc **(B)**, manganese **(C)**, nickel, molybdenum **(D)**, copper **(E)** and magnesium, potassium **(F)** were determined in entire plantlets and separated shoot tissue of 15-day-old wild type (wt, black bars) as well as 34-day-old *abci10-1* (grey bars) and *abci11-1* (white bars) mutant lines. Please note that for Ni and Mo in (D) shoot tissue of 20-day old seedlings was used. The respective metal content (n = 3 ± SD) is given relative to the level in wt, which was set to 100%. Data points with significant difference to wt according to Student’s t-test are indicated by *(p < 0.05) and ** (p < 0.01).

To follow a potential function of At-ABCI10 and At-ABCI11 in metal homeostasis, we determined metal contents in seedling and shoot tissues of wild type and *abci10-1*, *abci10-4, abci11-1* mutant lines (**Figure 6** and **Supplementary Figure 6E**). Whereas magnesium and potassium content did not change to a considerable extend in mutants when compared to wild type, the levels of transition metals exhibited pronounced alterations that were very similar in *abci10* and *abci11* lines. Prominent and most significant changes were observed for iron (Fe) and manganese (Mn), which play a central role in photosynthetic electron transport. All mutant lines *abci10-1*, *abci10-4*, and* abci11-1* displayed a two- to threefold increase in Fe-levels in shoots and entire seedlings when compared to wild type (**Figure 6A** and **Supplementary Figure 6E**). In contrast, Mn levels in mutant shoot tissue did not vary substantially, but in entire mutant plantlets were reduced to about half of the wild-type levels, indicating a more important role of Mn for root tissue (**Figure 6C**). The latter is supported by the finding that reduced root growth of *abci10-1*, *abci10-4*, and *abci11-1* can be partially rescued by additional Mn supply (**Supplementary Figures 6F**, **7C**). Fe addition to the medium, however, failed to prevent dwarfism of *abci10*, *abci11* knockout lines (**Supplementary Figure 7B**), well in line with the general Fe-overload measured. For zinc (Zn) and copper (Cu) as well as for nickel (Ni) and molybdenum (Mo), we observed increased levels in shoot tissue of *abci10*, *abci11* mutants, but a decrease of Zn and no change for Cu in entire seedlings (**Figures 6B, D, E** and **Supplementary Figure 6E**). Again, these findings indicate a severely impaired transition metal homeostasis in chloroplast-dominated shoot tissue that might lead to reduced uptake of Zn and also of Mn (see above) by mutant roots. The changes of metal content for *abci11-1* plantlets determined in this study—except for the decrease of Mn in complete seedlings—are well in line with the data previously published by Shimoni-Shor et al. (2010), who used separated shoot and root tissue. The observed iron overload in all mutant plants and the increase in shoot transition metals might explain the chlorotic and necrotic phenotypes, which are likely to be due to ROS stress generated by too many free metal ions.

**Figure 7:**
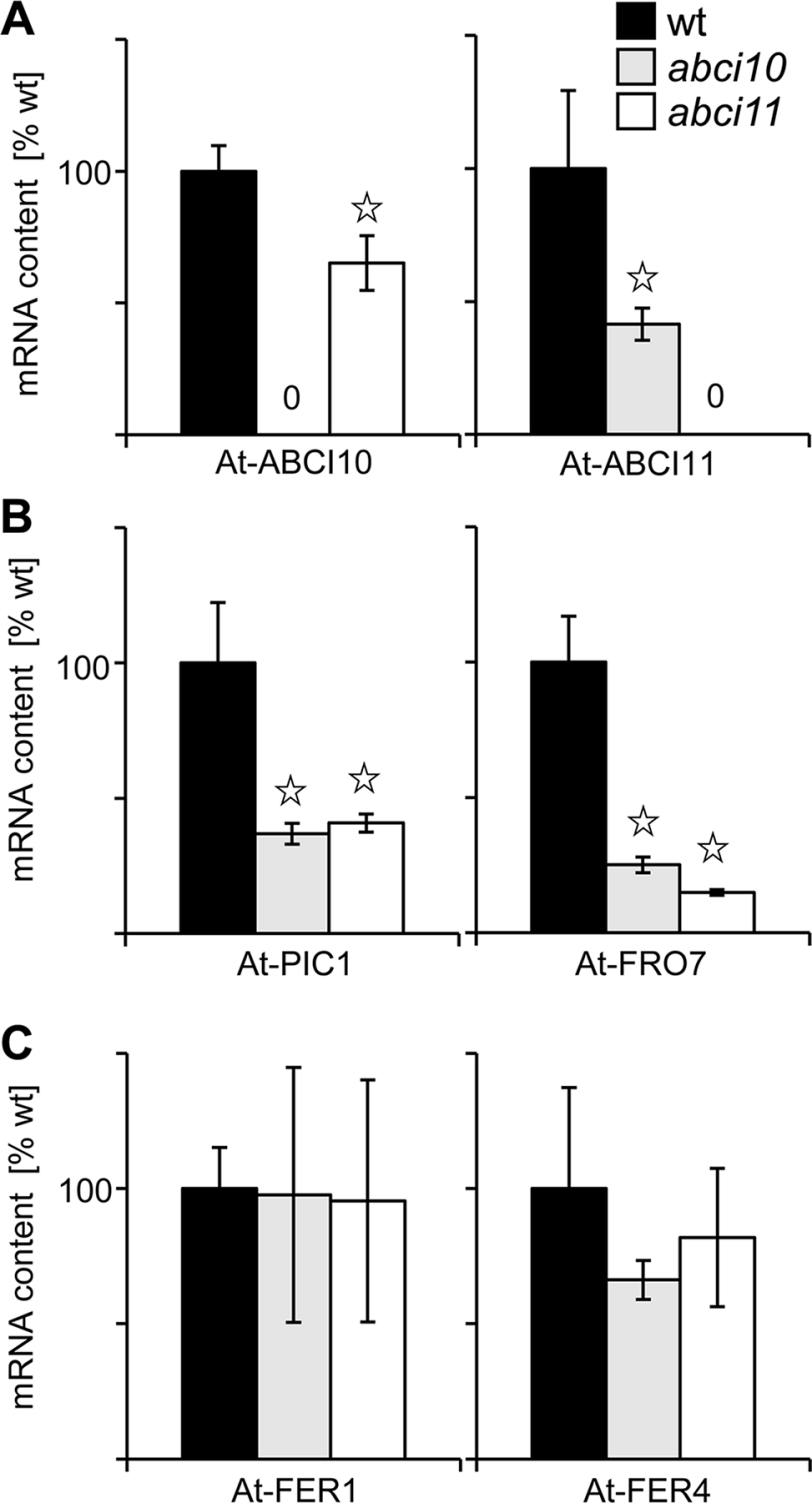
Knockouts of *At-ABCI10* and *At-ABCI11* show differential expression of chloroplast Fe-homeostasis and transport genes. Transcript levels were determined by qRT PCR on RNA isolated from 20-day-old seedlings of wild type (wt, black bars) as well as *abci10-1* (grey bars) and *abci11-1* (white bars) mutant lines. The mRNA content (n = 3 ± SD) of At-ABCI10, At-ABCI11 **(A)**, At-PIC1, At-FRO7 **(B)**, and At-FER1, At-FER4 **(C)** was normalized to 10.000 actin transcripts and calculated relative to the corresponding wild type, which was set to 100%. Data points with significant difference to wt according to Student’s t-test are indicated by * (p < 0.05).

To further link the function of ABCI10 and ABCI11 with iron in chloroplasts, we examined the gene expression levels of proteins related to Fe-homeostasis and transport in chloroplasts in *abci10-1* and *abci11-1* lines (**Figure 7**). First, we could demonstrate that expression of *At-ABCI11* is slightly down-regulated in *abci10-1* and *vice versa At-ABCI10* transcripts are decreased in *abci11-1* (**Figure 7A**). This behavior might indicate that ABCI10 and ABCI11 are functioning in separate pathways and cannot complement the function of each other. Moreover, transcripts of the Fe-uptake permease *PIC1* and the chloroplast ferric chelate reductase *FRO7* (**Figure 7B**) are significantly reduced in both mutant lines, pointing to a down-regulation of the potential reductive Fe-uptake pathway in plastids of *abci10-1* and *abci11-1* (compare Vigani et al., 2019). Transcripts of the major ferritin Fe-storage proteins *FER1* and *FER4* in the chloroplast stroma, however, remained at wild-type levels in both mutant lines (**Figure 7C**).

In conclusion, loss-of-function mutants of At-ABCI10 and At-ABCI11 are characterized by a de-regulated transition metal homeostasis, which is most pronounced for increased Fe- and decreased Mn-levels, both metals essential for photosynthesis. Although similar in their dwarf and for *abci10* also in albino appearance, *abci10-1* and *abci11-1* here behave opposite to knockout mutants of the chloroplast Fe-uptake permease PIC1, which shows no change in Fe-levels but a pronounced up-regulation of ferritin transcripts and proteins (Duy et al., 2007b).

### ABCI10 Appears to Interact With ABCI12 at the Chloroplast IE

**FIGURE 8:**
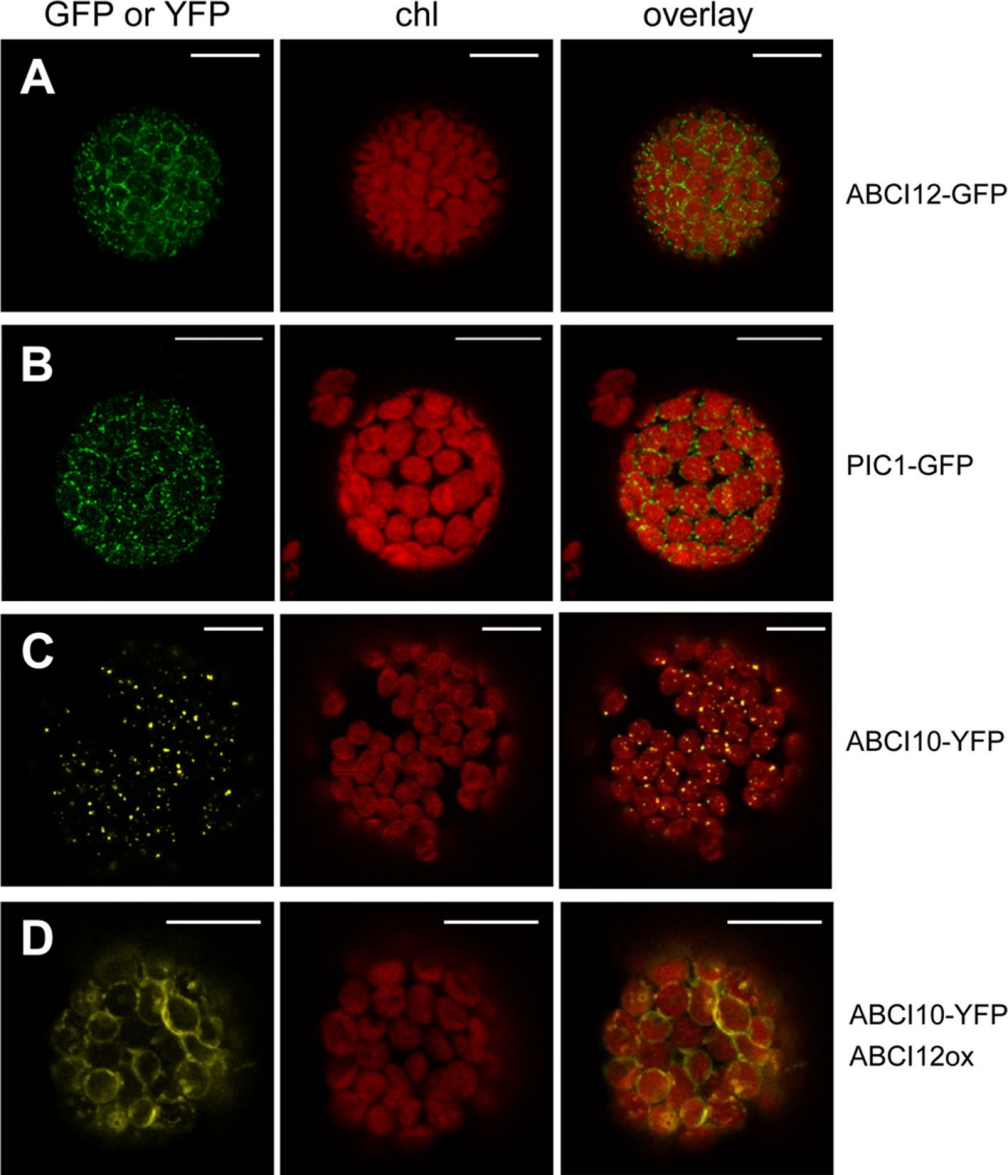
At-ABCI12 in the chloroplast envelope targets At-ABCI10 to the IE membrane. *Arabidopsis* leaf protoplasts were transiently transformed with constructs for At-ABCI12-GFP **(A)**, At-PIC1-GFP [chloroplast IE marker; (Duy et al. 2007b)] **(B)**, At-ABCI10-YFP **(C)**, as well as At-ABCI10-YFP when co-expressed with an At-ABCI12 construct **(D)**. Images show GFP- **(A,B)** and YFP-signals **(C,D)** (left), chlorophyll fluorescence (middle), as well as an overlay of both (right). Scale bar = 10 µm.

In order to identify potential IE membrane-intrinsic interaction partners for ABCI10 and ABCI11, we performed co-immunoprecipitation assays with the respective antisera on solubilized IE membrane vesicles from pea chloroplasts. Interestingly, the antiserum for At-ABCI10 precipitated a protein band around 50 kDa, which was identified to contain peptides of the pea ortholog to At-ABCI12 (**Supplementary Figure 8**). In contrast, this protein was not precipitated by α-ABCI11 or the respective pre-immune sera, indicating that the potential interaction with ABCI12 seems to be specific for ABCI10. For *Arabidopsis* At-ABCI12 (At3g21580), a chloroplast targeting peptide of 63 aa (ChloroP; Emanuelsson et al., 1999) and five α-helical transmembrane domains are predicted (Aramemnon database; Schwacke et al., 2003). To confirm the potential insertion of At-ABCI12 into the chloroplast envelope, we performed *in vivo* GFP targeting assays by transiently transforming isolated *Arabidopsis* protoplasts with At-ABCI12-GFP constructs (**Figure 8A**). Very similar to the chloroplast IE-intrinsic control At-PIC1-GFP (**Figure 8B**), At-ABCI12 targeted GFP fluorescence appeared around the chloroplast periphery, indicating insertion into the chloroplast envelope, most likely IE membranes. For a verification of the interaction of ABCI10 and ABCI12, we further co-transformed At-ABCI10-YFP and an over-expression construct for At-ABCI12. As observed previously (compare **Figure 2A**), At-ABCI10-YFP signals when transformed alone were punctuate at the periphery of chloroplasts (**Figure 8C**). In combination with overexpression of At-ABCI12 in the same protoplasts (co-transformation of both constructs), however, the YFP fluorescence of At-ABCI10 moved to a clear ring-like pattern around the chloroplast envelopes (**Figure 8D**). The same behavior was observed for co-expression of At-ABCI10-GFP and At-ABCI12-RFP, but not for At-ABCI11-GFP and At-ABCI12-RFP (**Supplementary Figure 9**).

Thus, fluorescence patterns of single and co-transformed At-ABCI10, At-ABCI11, and At-ABCI12 constructs confirm co-immunoprecipitation assays and indicate that indeed ABCI12 seems to interact with ABCI10 but not with ABCI11. Thereby, ABCI12 appears to anchor the potential ECF ATPase-subunit ABCI10 to the IE membrane of chloroplasts.

### ABCI12: Subunit T of a Potential Chloroplast ECF ABC Transporter

**FIGURE 9:**
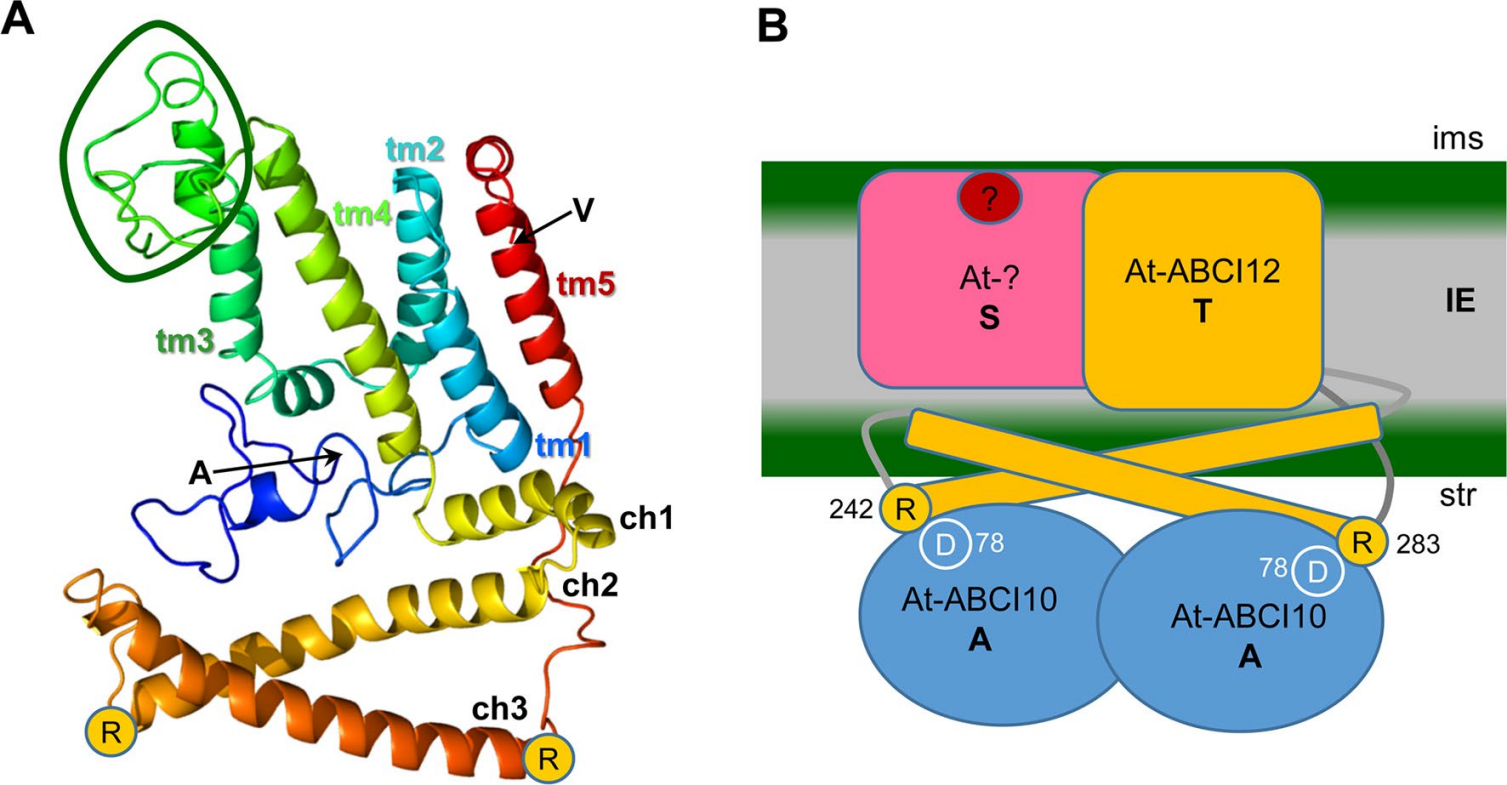
ABCI12 as T subunit of a chloroplast ECF ABC transporter. **(A**) The secondary structure of mature At-ABCI12 (79% modelled at >90% confidence) was predicted by Phyre^2^ (Kelley et al. 2015) in comparison to the structure of four bacterial ECF transporter complexes (templates 5js7, c4huqT [FolT folate transport], c4rfsT [panthothenate transport], c5x3xq [CbiMQO Co-transport]. N- and C-terminal amino acids of the mature ABCI12 (alanine and valine, respectively) as well as the conserved arginine residues (R) at the end of ch2 and ch3 are indicated. Please note that the structures of the N-terminus (blue ribbon) and of the loop between tm3 and tm4, which is specific for plant and cyanobacterial ABCI12 relatives (green circle), are predicted with low confidence, whereas helices are strongly supported by the model. ch1-3, coupling α-helices; tm1-5, transmembrane α-helices 1-5. **(B)** According to the working hypothesis, At-ABCI12 (orange) in the IE membrane would represent the energy transducing subunit T of a chloroplast ECF transporter AAT module. ch2 and ch3 of ABCI12 could bind with R_242_ and R_283_ to conserved aspartate residues (D_78_) in the groove of an At-ABCI10 ATPase homodimer (AA subunit, blue) at the inner leaflet of the IE. The membrane-intrinsic substrate-binding subunit S (pink) as well as the substrate (red dot), however, are still unknown. ims, intermembrane space; str, stroma.

The mature At-ABCI12 protein is expected to be 328 aa long with a size of about 36 kDa. In UniProt/InterPro databases (accession Q944H2), At-ABCI12 is annotated to be an ABC/ECF transporter transmembrane component (IPR003339 family). Further, the protein was already mentioned as “plant T protein,” belonging to a plant-specific ECF transporter T subunit with similarity to cyanobacterial proteins (Eitinger et al., 2011). The crystal structures of CbiQ2 and EcfT in the complexes CbiMQO (group I, Co-uptake; Bao et al., 2017) and ECF-FolT (group II, folate transport; Swier et al., 2016) reveal details in structure function relations of T subunits and therefore were selected for comparison with At-ABCI12. In addition, we choose NikQ (group I, Ni-transport) and BioN (group I, biotin transport), the latter only with four α-helical membrane domains (**Supplementary Figure 10**). Here, At-ABCI12 and its ortholog Os-ABCi8 from rice show structural similarity to T subunits with 5 α-helical transmembrane domains, namely, NikQ, CbiQ2, and EcfT from ECF complexes for Ni, Co, and folate transport (NikMNQO, CbiMNQO, and FolT, respectively; Rodionov et al., 2006; Neubauer et al., 2009; Rodionov et al., 2009). Like for ABCI10 and ABCI11, orthologs of ABCI12 are found in the green lineage in dicots, monocots, mosses, green algae, and cyanobacteria (**Supplementary Figure 11**). However, ABCI12 relatives appear to be absent in *Gloeobacter*, i.e., cyanobacteria without thylakoid membrane systems. In comparison to non-photosynthetic prokaryotes, it becomes evident that plant and cyanobacterial T proteins contain an additional stretch—about 35 amino acids for At-ABCI12—between transmembrane helix 3 and 4 (**Figure 9A**, **Supplementary Figures 10**, **11**; compare Eitinger et al., 2011).

Structural modeling of the mature At-ABCI12 (Phyre2; Kelley et al., 2015) gives a structure prediction also with five transmembrane helices known for most EcfT proteins (group II) and many T subunits of group I transporters (Eitinger et al., 2011) with considerable confidence (**Figure 9A**). Common and conserved for all T proteins of ECF transporters are the two coupling helices with the highly conserved X-R-X motifs, crucial for interaction with the negatively charged residues in the groove of the ATPase AA dimer (compare **Figure 1**) at the cytosolic face of the membrane (Neubauer et al., 2009; Rempel et al., 2019). In At-ABCI12, these conserved arginines can be found at the C-terminal end of each of the coupling helices ch2 (R_242_) and ch3 (R_283_) (**Figure 9**). Since ABCI12 most likely inserts into the IE membrane of chloroplasts, we propose that these two coupling helices are facing to the stroma and thereby can mediate interaction with the groove of a potential ABCI10 AA dimer (**Figure 9B**).

## Discussion

In our study on potential metal transporters of prokaryotic origin in *Arabidopsis* chloroplasts, we identified the soluble NBD ABC-transporter subunits At-ABCI10 and At-ABCI11. By *in vivo* GFP-targeting and immunoblot analysis, we could unequivocally show that both proteins are chloroplast intrinsic. Thereby, we confirmed previous results on ABCI11/NAP14 in *Arabidopsis* and rice (Shimoni-Shor et al., 2010; Zeng et al., 2017). Our *in vivo* GFP-targeting assays in the homologous *Arabidopsis* protoplast system appeared to allow to distinguish signal distribution within different regions of the chloroplast stroma—i.e., soluble for the control FSD1 or membrane attached for ABCI11 (plastoglobuli) and ABCI10 (envelope). However, interpretation of data and biological relevance of these observations requires a second, independent approach such as immunoblot analysis. The latter revealed to our surprise that both ABCI10 and ABCI11 appeared to be strongly attached to chloroplast IE membranes. For At-ABCI11 *in vivo* GFP-targeting assays in addition point to a possible attachment to plastoglobuli (PG), which still has to be verified. On the one hand, transient overexpression of fluorescent-tagged proteins could result in ectopic, non-endogenous localization of the proteins. On the other hand, PG membranes might co-purify with the IE membrane vesicles during purification steps. Further, it should also be noted that a number of studies on plastoglobule proteomics did not find ABCI11 (Ytterberg et al., 2006; Lundquist et al., 2012; van Wijk and Kessler, 2017). In proteomics of chloroplast envelopes, ABCI11 was also not experimentally detected, but only annotated to the IE (Ferro et al., 2010). Thus, if the sub-organellar distribution of the ABCI11 protein is only at plastoglobuli or maybe at both IE and PG lipid layers, still has to be clarified in the future. Here, detailed suborganellar immuno-localization by electron microscopy techniques and the identification of ABCI11-interacting proteins would help to unveil the precise localization and function of ABCI11 proteins in chloroplasts. Depending on the interaction partner, At-ABCI11/NAP14 might either represent an ATPase NBD-domain subunit of a canonical prokaryotic-type ABC transporter or function as non-ABC transporter, organelle-intrinsic NAP protein. Within the plant ABC-transporter inventory in subfamily I (Verrier et al., 2008), the TGD1-3 complex—NBD-TMD-SBP subunit arrangement—as well as the NBD-TMD dimers ABCI1-ABCI2 and ABCI17-ABCI16 are examples for a canonical prokaryotic-type ABC importer assembly (for details on functions see Xu et al., 2003; Lu et al., 2007; Rayapuram et al., 2007; Huang et al., 2010; Xu et al., 2010; Roston et al., 2012; Belal et al., 2015; Dong et al., 2017; Wang et al., 2019). The NBD ATPase ABCI6 for FeS cluster biogenesis in chloroplasts, however, interacts with ABCI7, ABCI8, which are soluble proteins that do not belong to an ABC transporter assembly (Xu and Möller, 2004). To our knowledge, no more single transmembrane subunits of either canonical TMD- or ECF T-type with still unknown partners are annotated in the plant ABC transporter superfamily (Verrier et al., 2008). Therefore, an interaction of ABCI11 with protein partners similar to ABCI7, ABCI8 for FeS cluster biogenesis seems to be most likely. Thus, ABCI11/NAP14 appears to group into the “non-intrinsic ABC protein” NAP family and bind to a still unknown partner protein at plastoglobuli and/or IE membranes. We can, however, not include that some still anonymous membrane-intrinsic proteins for interaction with ABCI11 are present in the chloroplast proteome.

For At-ABCI10 instead, interpretation of the data is more straightforward and points to a strong attachment to the IE membrane from the stroma side. i) At-ABCI10 was the only protein of the three examined in this study, for which peptides have been experimentally detected in purified envelopes from *Arabidopsis* (Froehlich et al., 2003), envelope preparations from maize (Bräutigam et al., 2008), and IE membranes from pea (Gutierrez-Carbonell et al., 2014). ii) No signals of fluorescence-tagged At-ABCI10 have been associated with plastoglobuli. iii) Attachment of ABCI10 to IE membranes seems to be a bit stronger than for ABCI11. iv) The presence of the conserved Q-helix motif in ABCI10, which indicates that in the *Arabidopsis* ABCI family only ABCI10 represents a potential ATPase A subunit of an ECF AAT module. v) Identification of ABCI12 as potential ECF T-subunit interaction partner and IE membrane-anchor for ABCI10.

The loss-of-function of ABCI10 as well as of ACBI11 in *Arabidopsis*, however, severely impacts plant growth and development as documented by the dwarf, albino appearance of seedlings as well as partial embryo lethality and the fact that homozygous lines are unable to reproduce. Furthermore, chloroplast and in particular thylakoid biogenesis are defect. Since on the chloroplast ultrastructural level and in their completely albino appearance, phenotypes of *abci10* knockouts appear to be more severe than those of *abci11*, a function of both proteins in different pathways is plausible. This hypothesis is further supported by the finding that gene expression in seedlings of both *At-ABCI10* and *At-ABCI11* is not up-regulated for complementation in the respective knockout mutants. However, although somewhat different in their suborganellar distribution, both At-ABCI10 and At-ABCI11 are clearly associated with a function in cellular metal homeostasis. The latter also has been documented previously for mutants of rice and *Arabidopsis* ABCI11 (Shimoni-Shor et al., 2010; Zeng et al., 2017). In the stroma of *abci10* and *abci11* mutant plastids, we detected accumulations of electron-dense material, which resemble that of ferritin protein clusters found in *pic1* knockout plastids (compare Duy et al., 2007b). Thus, in addition to deregulated metal content and reduced transcripts of *PIC1* and *FRO7* genes for chloroplast Fe-uptake in *abci10*, *abci11* mutant seedlings, these ferritin-like clusters in *abci10* and *abci11* plastids point to unbalanced chloroplast metal homeostasis. Different to the strong increase of ferritin transcript and protein in *pic1*, gene expression of ferritin, however, appeared not to be regulated in *abci10* and *abci11*. Furthermore, also increased Fe-levels in *abci10* and* abci11*, which are absent in *pic1* (Duy et al., 2007b), point to different cellular regulation in response to the loss of PIC1 and ABCI10, ABCI11. Interestingly, *At-ABCI10*, *At-ABCI11*, and *At-ABCI12* genes show a very similar expression pattern when compared to *At-PIC1*. For all four genes, transcripts peak in green shoot tissues and are almost absent in roots (see developmental map at *Arabidopsis* eFP browser; https://bar.utoronto.ca/efp_arabidopsis/cgi-bin/efpWeb.cgi). The absolute transcript levels for *At-ABCI10*, *At-ABCI11*, and *At-ABCI12*, however, are about 10-fold less than for *At-PIC1*. The partial rescue of dwarf root growth in *abci10 *and *abci11* mutants by Mn supplementation also indicates a role for both proteins in metal homeostasis. A pronounced decrease of Mn as well as of Zn was observed only in entire *abci10* and *abci11* seedlings but not in separated shoot tissue. Thus, our findings indicate a severely impaired transition metal homeostasis in chloroplast-dominated shoot tissue. This implies that in response to transition metal overload in shoots, and in particular to the prominent overall increase of Fe, *abci10* and *abci11* mutants might down-regulate root metal acquisition systems. Thus, it is likely that the root growth rescue by Mn is due to secondary effects and not directly linked to a function of ABCI10 and ABCi11 in root plastids. Most likely, ABCI10 (IE attached) and ABCI11 (PG associated) due to their distinct distribution in the chloroplast stroma and their potential assembly with separate protein complexes fulfill different tasks. However, both are crucial for transition metal homeostasis in chloroplasts and thereby closely linked to photosynthetic performance. For the latter, impact of ABCI10 seems to be larger than that of ABCI11, since the albino phenotype of *abci10* knockouts is more severe.

The observed interaction of ABCI10 with ABCI12 at the chloroplast IE membrane suggests that both proteins are part of a prokaryotic-type ECF ABC-transporter. Here, ABCI10 would represent the ATP-binding subunit A, and ABCI12 the membrane-intrinsic, energy-transducing subunit T (**Figure 9B**). According to the working models developed for the prokaryotic CbiMNQO (group I ECF for Co-uptake; Bao et al., 2017) and FolT2 (group II, folate import; Swier et al., 2016), the coupling helices ch2 and ch3 of plant ABCI12 could anchor an ABCI10 dimer at the inner leaflet of the IE membrane (**Figure 9B**). In general, hydrophobic and hydrophilic interactions between the coupling helices of ECF T-subunits and the groove of AA dimers are described (Swier et al., 2016; Bao et al., 2017). In particular, ionic interactions *via* the two conserved positively charged arginine motifs at the end of ch2 and ch3 from the T component and the negatively charged aspartate residues in the helical subdomain of each A protein contribute to the AAT module assembly (Rempel et al., 2019). For the mature ABCI12 protein, these residues are represented by arginine 242 (ch2) and 283 (ch3), the conserved aspartate in the mature ABCI10 sequence is at position 78 (**Figure 9B**). Thereby, the observed unusually strong attachment of ABCI10 to IE membranes could be explained. If ABCI10 functions as homodimer or maybe in combination with another ATPase A subunit still has to be clarified. However, since ABCI10 represents the only ABC ATPase with the ECF transporter specific Q-helix motif in *Arabidopsis*, a function as homomeric AA component is most likely. Furthermore, ABCI10 proteins do not contain the C-terminal α-helical stretch (compare **Figure 1**), which is mandatory for ECF group II but only optional for group I transporters (Eitinger et al., 2011; Rempel et al., 2019). Thus, the potential ABCI10/ABCI10-ABCI12 AAT module in the IE membrane of chloroplasts, most likely belongs to group I ECF importers.

In bacteria, these group I ECF transporters are described to import divalent Co or Ni metal ions (CbiMNQO, NikMNQO) as well as metabolites like biotin (BioMNY), methyl-thioadenosine or precursors for queuosine, methionine, thiamine, or cobalamin (Rodionov et al., 2009; Eitinger et al., 2011). Transport specificity is defined by the corresponding substrate-binding protein S, whose origin and features are still enigmatic for the potential chloroplast ABCI10/ABCI10-ABCI12 module. In general, a chloroplast ECF-complex mediated uptake of divalent metal ions for photosynthetic electron transport or import of biotin, which is synthesized in mitochondria but central for *de novo* fatty acid biosynthesis in chloroplasts, is probable. Since *abci10* knockout lines show a strong albino phenotype, similar to that of loss-of-function lines for the chloroplast Fe-uptake permease PIC1 (Duy et al., 2007b; Duy et al., 2011; Gong et al., 2015), it is however tempting to speculate that ABCI10 in chloroplasts is involved in transition metal uptake as well. Thereby, the potential ABCI10/ABCI10-ABCI12 module would provide a bypass for PIC1 in IE membranes, which, however, has a distinct impact on cellular metal homeostasis. Furthermore, PIC1 (Duy et al., 2007b) and ABCI12 orthologous proteins seem to be absent in the thylakoid-less green algae *Gloeobacter* pointing to a transport function closely linked to thylakoid membrane processes such as photosynthetic electron transport that requires a high amount of transition metals (Fe, Mn, Cu). In addition, the plant and cyanobacterial specific amino acid stretch between tm3 and tm4 of T subunits (compare **Figure 9B**; Eitinger et al., 2011) indicates a specification linked to performance of oxygenic photosynthesis. Since tm3 is described to contribute to conformational flexibility between membrane-intrinsic and coupling domains of ECF T-subunits and together with the other hydrophobic tms is involved in interaction with the membrane-intrinsic S component (Rempel et al., 2019), this “photosynthesis”-specific stretch in ABCI12 proteins might define the still unknown substrate-specificity of the potential chloroplast AAT module. Because specificity of transport proteins for divalent metal ions in general is low (Kobayashi and Nishizawa, 2012), we propose that ABCI10/ABCI10-ABCI12 might be involved in chloroplast uptake of Fe and Mn ions.

Involvement of ABCI10 and ABCI12 in chloroplast biotin uptake seems rather unlikely, because the five transmembrane structure predicted for ABCI12 is not common for BioN T subunits in ECF biotin transporters. Further, since the chloroplast stroma is the only site for *de novo* fatty acid synthesis in plant cells, disruption of biotin import essential for the first step of this pathway would most likely lead to complete embryo lethality rather than to the chlorotic and dwarf appearance and only partial embryo lethality observed in *abci10* knockouts. Indeed, strict embryo abortion is observed in knockout mutants of the biotin-carboxyl carrier protein BCCP2 (Li et al., 2011), which delivers biotin for carboxylation in the heteromeric acetyl-CoA carboxylase complex in the chloroplast stroma (Li-Beisson et al., 2013).

In summary, it is tempting to speculate that ABCI10 as homodimer and ABCI12 in the IE membrane can form an AAT energy-coupling module of a novel, chloroplast ECF ABC importer that most likely has a prokaryotic origin and is involved in transition metal uptake. A conclusion on assembly and transport specificity of a potential group I ECF transport, mediated by an ABCI10/ABCI10-ABCI12 module however, is only possible with the still lacking identification of the substrate-binding component(s) and direct functional as well as interaction assays for such a complex.

## Data Availability Statement

All datasets generated for this study are included in the manuscript/**Supplementary Files**.

## Author Contributions

JP, YL, and KP conceived and designed experiments. LV and JP characterized mutant plants and phenotypes, performed GFP-targeting assays, and analyzed metal contents. LV conducted co-immunoprecipitation assays and qRT-PCR analysis. RS purified recombinant proteins and contributed to immunoblot analysis and co-transformation of protoplasts. CL contributed to phenotyping of mutant lines. KP performed structural and sequence analysis of ABCI proteins and wrote the manuscript together with JP.

## Funding

This work was funded by the DFG (Deutsche Forschungsgemeinschaft) grants PH73/3-2, -3 to KP, a Human Frontier Science Program long-term postdoctoral fellowship to JP, and in part was supported by the National Research Foundation of Korea (NRF) grant (2018R1A2A1A05018173) funded by the Korean government (Ministry of Science and ICT) to YL.

## Conflict of Interest

The authors declare that the research was conducted in the absence of any commercial or financial relationships that could be construed as a potential conflict of interest.
