# Supplementary material for: A Novel Prokaryote-Type ECF/ABC Transporter Module in Chloroplast Metal Homeostasis
Source: Front Plant Sci. 2019 Oct 29;10:1264. doi: 10.3389/fpls.2019.01264 (PMC6828968; doi:10.3389/fpls.2019.01264)
Supplement: Supplementary file 1 [file Image_1.pdf]

Figure S1

A

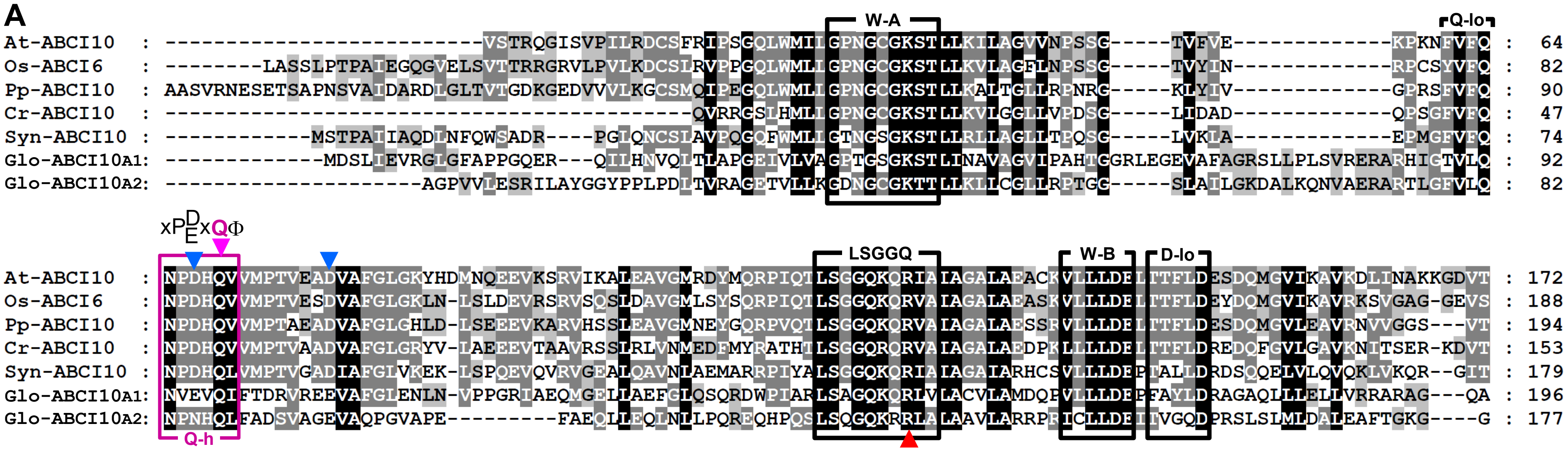

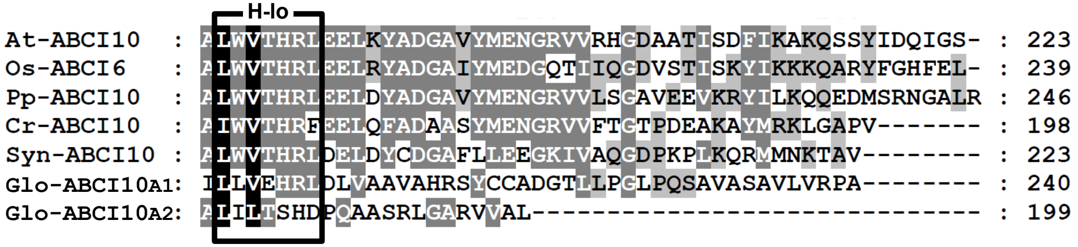

B

	At	Os	Pp	Cr	Syn	GloA1	GloA2
At-ABCI10	100%						
Os-ABCI6	63%	100%					
Pp-ABCI10	55%	60%	100%				
Cr-ABCI10	51%	46%	49%	100%			
Syn-ABCI10	45%	48%	44%	42%	100%		
Glo-ABCI10A1	22%	25%	22%	20%	27%	100%	
Glo-ABCI10A2	21%	23%	22%	24%	23%	25%	100%

FIGURE S1 | ABCI10 is conserved among plants and cyanobacteria. **(A)** Amino acid sequence alignment of ABCI10 relatives. Different organisms, gene numbers and NCBI Reference Sequences of Gen-Pept entries are given in brackets. Please note that for all plant proteins the predicted mature sequences according to ChloroP (Emanuelsson et al., 1999) are depicted. At-ABCI10 (*Arabidopsis thaliana*, At4g33460, NP_195072), Os-ABCI6 (*Oryza sativa [japonica]*, Os01g56400, BAD52721), Pp-ABCI10 (*Physcomitrella patens*, XP_024370958), Cr-ABCI10 (*Chlamydomonas reinhardtii*, XP_001703542), Syn-ABCI10 (*Synechocystis PCC 6803*, gene sll1623, WP_010872403), Glo-ABCI10A1 and A2 (*Gloeobacter violaceus*, NP_925000). The conserved motifs Walker A, Walker B, Q-loop, D-loop, and H-loop (black boxes) form the nucleotide binding sites and the ABC-transporter signature motif LSGGQ is found in the helical subdomain. ABCI10 relatives contain the ECF specifc Q-helix motif (X-P-D/E-X-Q-Φ: X is any, Φ a hydrophobic amino acid) indicated by a purple box (compare **Figure 1A**). **(B)** Amino acid identities in % of proteins depicted in **(A)**.
