# Supplementary material for: A Novel Prokaryote-Type ECF/ABC Transporter Module in Chloroplast Metal Homeostasis
Source: Front Plant Sci. 2019 Oct 29;10:1264. doi: 10.3389/fpls.2019.01264 (PMC6828968; doi:10.3389/fpls.2019.01264)
Supplement: Supplementary file 2 [file Image_2.pdf]

Figure S2

A

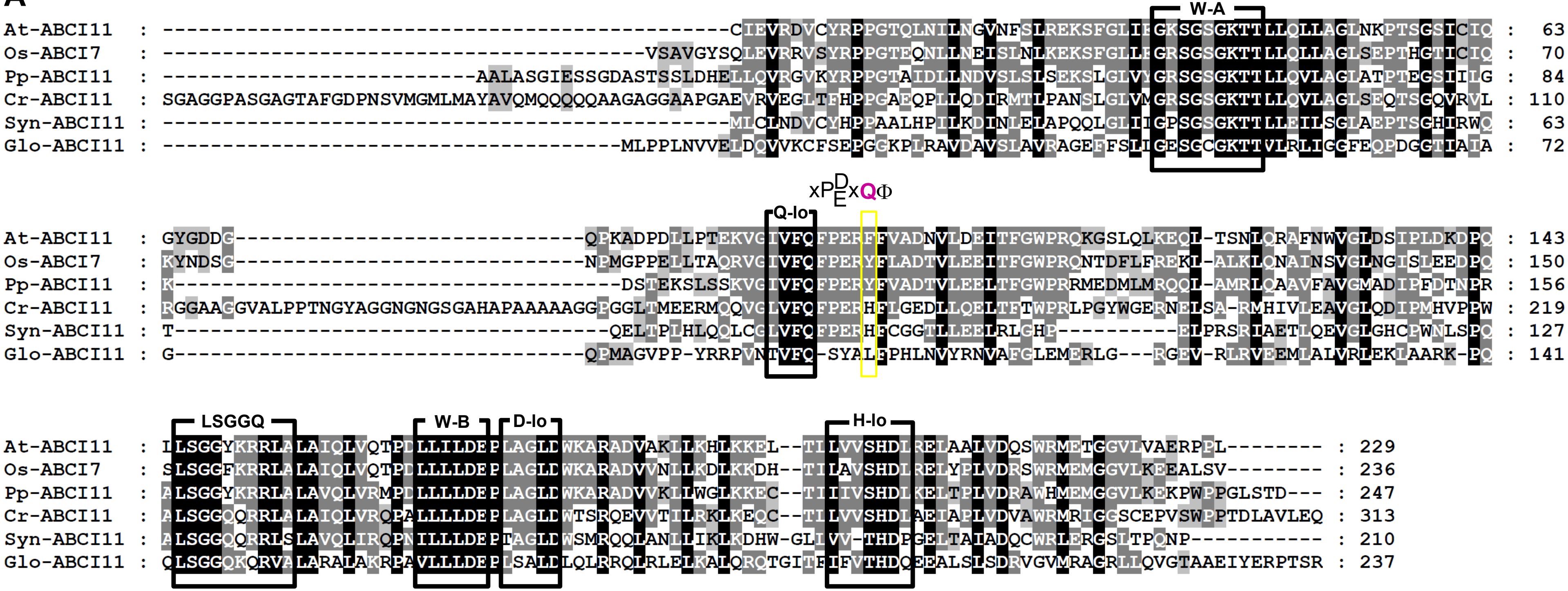

B

	At	Os	Pp	Cr	Syn	Glo
At-ABCI11	100%					
Os-ABCI7	65%	100%				
Pp-ABCI11	52%	57%	100%			
Cr-ABCI11	34%	36%	37%	100%		
Syn-ABCI11	41%	38%	38%	32%	100%	
Glo-ABCI11	29%	29%	26%	19%	24%	100%

FIGURE S2 | ABCI11 is conserved among plants and cyanobacteria. **(A)** Amino acid sequence alignment of ABCI11 relatives. Different organisms, gene numbers and NCBI Reference Sequences of GenPept entries are given in brackets. Please note that for all plant proteins the predicted mature sequences according to ChloroP (Emanuelsson et al., 1999) are depicted. At-ABCI11 (*Arabidopsis thaliana*, At5g14100, NP_196914), Os-ABCI7 (*Oryza sativa [japonica]*, Os11g29850, ABA93815), Pp-ABCI11 (*Physcomitrella patens*, XP_024369396), Cr-ABCI11 (*Chlamydomonas reinhardtii*, PNW71978), Syn-ABCI11 (*Synechocystis PCC 6803*, gene slr0354, WP_010873396), Glo-ABCI11 (*Gloeobacter violaceus*, WP_011141404). The conserved motifs Walker A, Walker B, Q-loop, D-loop, and H-loop (black boxes) form the nucleotide binding sites. The ABC-transporter specific helical subdomain contains the signature motif LSGGQ. The ECF-specific Q-helix motif X-P-D/E-X-Q-Φ is absent in ABCI11 proteins because the conserved glutamine residue is substituted by tyrosine, phenylalanine, lysine or histidine residues (yellow box, compare **Figure 1A**). **(B)** Amino acid identities in % of proteins depicted in **(A)**.
