# Supplementary material for: A Novel Prokaryote-Type ECF/ABC Transporter Module in Chloroplast Metal Homeostasis
Source: Front Plant Sci. 2019 Oct 29;10:1264. doi: 10.3389/fpls.2019.01264 (PMC6828968; doi:10.3389/fpls.2019.01264)
Supplement: Supplementary file 3 [file Image_3.pdf]

Figure S3

A

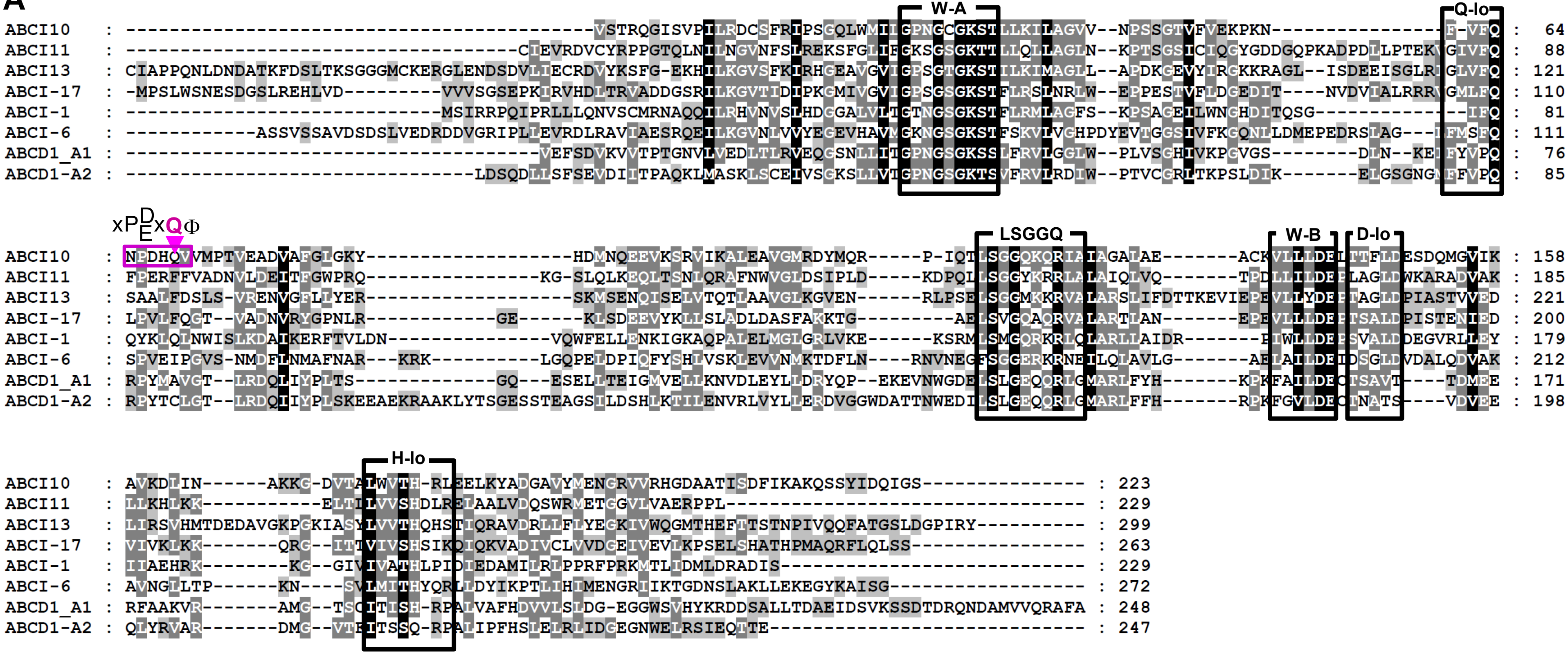

B

	I10	I11	I13	I17	I1	I6	D1-A1	D1-A2
ABCI10	100%							
ABCI11	24%	100%						
ABCI13	21%	21%	100%					
ABCI17	21%	19%	28%	100%				
ABCI1	18%	19%	15%	17%	100%			
ABCI6	15%	21%	16%	15%	15%	100%		
ABCD-A1	15%	16%	14%	19%	14%	15%	100%	
ABCD-A2	13%	14%	13%	16%	15%	12%	37%	100%

FIGURE S3 | ABCI family proteins from *Arabidopsis* with NBD binding domains. **(A)** Amino acid sequence alignment of *Arabidopsis* prokaryotic-type ABCI proteins with NBD binding domains (for gene codes see subfamily I in (Verrier et al., 2008)) The NBD-binding domains of the full-size eukaryotic-type ABC transporter ABCD1 (At1g54350) are for comparison. The conserved motifs Walker A, Walker B, Q-loop, D-loop, and H-loop (black boxes) form the nucleotide binding sites. The ABC-transporter specific helical subdomain contains the signature motif LSGGQ. Please note that the ECF specific Q-helix motif X-P-D/E-X-Q-Φ (purple box) is only present in ABCI10 (compare **Figure 1A, Figure S1, Figure S2**).
