# Supplementary material for: A Novel Prokaryote-Type ECF/ABC Transporter Module in Chloroplast Metal Homeostasis
Source: Front Plant Sci. 2019 Oct 29;10:1264. doi: 10.3389/fpls.2019.01264 (PMC6828968; doi:10.3389/fpls.2019.01264)
Supplement: Supplementary file 4 [file Image_4.pdf]

Figure S4

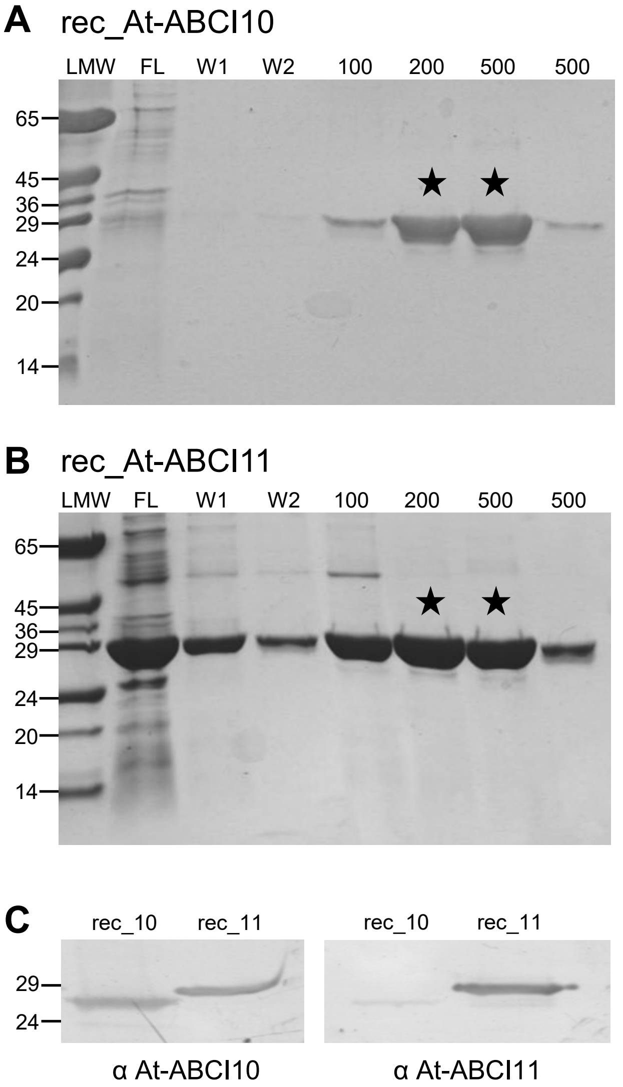

FIGURE S4 | Production of recombinant At-ABCI10 and At-ABCI11 proteins.
Ni-NTA sepharose purification of rec_At-ABCI10-[6His] **(A)** and rec_At-ABCI10[6His] **(B)** over-expressed in *E.coli* cells as described in Materials and Methods. 10µl of each protein fraction in buffer A (50 mM NaPP, pH 8.0, 100 mM NaCl, 2 mM β-mercaptoethanol, 8 M urea) were separated by SDS-PAGE and Coomassie stained. Purification fractions are as follows: FL, flow through of column; W1, W2, wash of column; 100, 200, 500, elution with 100-500 mM imidazole in buffer A. LMW, low molecular weight marker. Asterisks indicate purified proteins used for generation of antisera. Please note that rec_At-ABCI10 runs at 28kDa, and rec_At-ABCI11 at 29kDa. **(C)** 25 mg of each recombinant protein At-ABCI10 and At-ABCI11 purified in **(A), (B)** were separated by SDS-PAGE and subjected to immuno-blot analysis to check for cross-reaction with the generated antisera α-At-ABCI10 (left) and α-At-ABCI11 (right). Please note that α-At-ABCI10 cross-reacts with the rec_At-ABCI11 protein. Numbers indicate molecular mass of proteins in kDa.
