# Supplementary material for: A Novel Prokaryote-Type ECF/ABC Transporter Module in Chloroplast Metal Homeostasis
Source: Front Plant Sci. 2019 Oct 29;10:1264. doi: 10.3389/fpls.2019.01264 (PMC6828968; doi:10.3389/fpls.2019.01264)
Supplement: Supplementary file 5 [file Image_5.pdf]

Figure S5

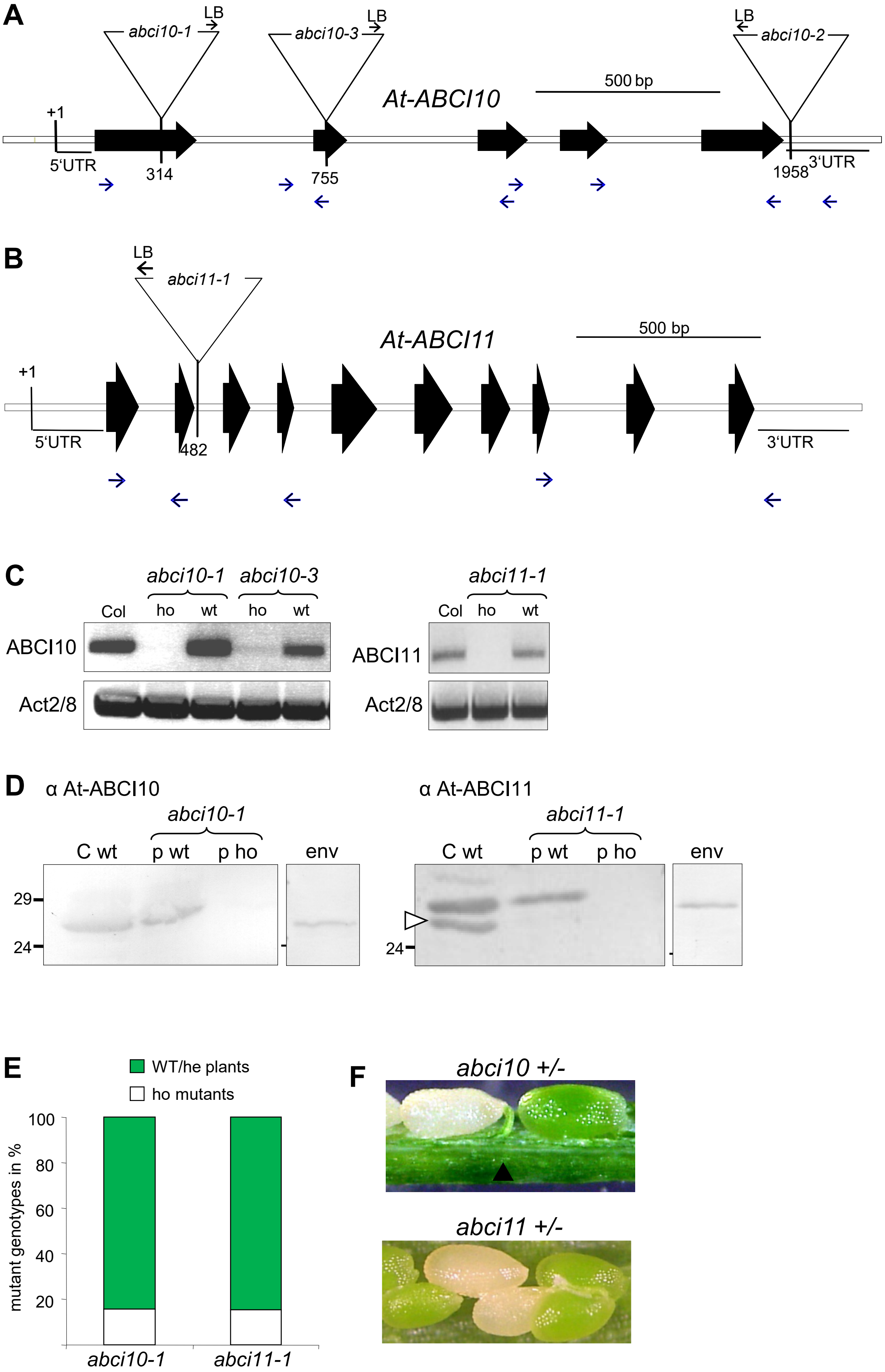

A
LB
abci10-1
LB
abci10-3
LB
abci10-2
At-ABCI10
500 bp
+1
5'UTR
314
755
1958
3'UTR
B
LB
abci11-1
At-ABCI11
500 bp
+1
5'UTR
482
3'UTR
C
abci10-1
abci10-3
abci11-1
Col
ho
wt
ho
wt
Col
ho
wt
ABCI10
ABCI11
Act2/8
Act2/8
D
α At-ABCI10
α At-ABCI11
abci10-1
abci11-1
C wt
p wt
p ho
env
C wt
p wt
p ho
env
29
24
24
E
WT/he plants
ho mutants
100
80
60
40
20
mutant genotypes in %
abci10-1
abci11-1
F
abci10 +/-
abci11 +/-

FIGURE S5 | Characterization of the T-DNA mutants *abci10-1*, *abci10-3*, and *abci11-1.*
(A) The gene *At-ABCI10* (At4g33460) contains 5 exon regions (black arrows). T-DNA insertions for the mutant lines *abci10-1*, *abci10-3*, *abci10-2* are at base pairs (bp) 314, 755 and 1958, respectively. **(B)** The gene *At-ABCI11* (At5g14100) contains 10 exon regions (black arrows). T-DNA insertion for the mutant line *abci11-1* is at position 482. The positions of primers used for genotyping as described below, are indicated by blue arrows. UTR, untranslated regions. Genomic DNA of the heterozygous T-DNA insertion lines *abci10-1* (SALK_027278), *abci10-3* (GABI_969_D10), and *abci11-1* (SALK_116866) was screened by PCR genotyping (not shown). *At-ABCI10* or *At-ABCI11* gene-specific primers in combination with T-DNA–specific left border primers generated fragments of 680 bp (*abci10-1*), 566 bp (*abci10-3*), and 487 bp (*abci11-1*) on heterozygous and homozygous plants. To identify plants with the T-DNA insertion in both alleles of *At-ABCI10* or *At-ABCI11* respectively, we used gene-specific primers flanking the predicted T-DNA insertion sites. DNA from homozygous, mutated *abci10* or *abci11* gave no amplification product, whereas the amplified regions on wild-type and heterozygous DNA were 622 bp (*abci10-1*), 626 bp (*abci10-3*), and 517 bp (*abci11-1*). To verify PCR products and T-DNA insertion sites, all amplified DNA fragments were sequenced. **(C)** RT-PCR on cDNA from Col-0 as well as segregated homozygous and wild-type plants of the mutants *abci10-1*, *abci10-3*, and *abci11-1* as described in **(A)**, **(B)**. Primer pairs specific for *At-ABCI10* and *At-ABCI11* only in wild-type plants amplifed products of 347 bp and 330 bp, respectively. Showing that *abci10-1*, *abci10-3*, and *abci11-1* homozygous lines are knockouts without RNA of the respective genes. A PCR product of actin (435 bp) was used as control. **(D)** Immunoblots on isolated chloroplasts (C) and total protein extracts (p) from homozygous *abci10-1*, *abci11-1* and the corresponding wild-type lines (wt) in *Arabidopsis* as well as on envelope membranes (env) purified from *Arabidopsis* chloroplasts. On each lane 20 µg of proteins or 10µg for env were separated by SDS-PAGE. The antiserum for At-ABCI10 (left panel), specifically detects the mature ABCI10 protein (27 kDa) only in wt, showing that the *abci10-1* knockout is also consistent at the protein level. α-At-ABCI11 (right panel) as well decorates the mature AtABCI11 (28.5 kDa) only in wt, confirming the knockout of At-ABCI11 in *abci11-1*. Please note that in chloroplasts of wt, a second band of about 28 kDa appears (triangle), which is absent in total protein extracts of wt and ho. Thus, it is tempting to speculate that the lower signal in the observed double band for α At-ABCI11 in pea IE membranes (compare **Figure 3A**) as well originates from unspecific interaction with an unknown, chloroplast-intrinsic ABCI protein. **(E)** Segregation analysis of homozygous (ho) progeny from *abci10-1*, *abci11-1* lines. PCR-genotyping of the *abci* mutant lines described in **(A)**, **(B)** reveals that ho progeny only segregates with a probability of 15.6 % for *abci10-1* (n = 3922) and 15.4 % for *abci11-1* (n = 3692). This non-Mendelian segregation of both mutants indicates possible defects in gametophyte and/or embryo and seed developement. **(F)** Empty seed coats with aborted embryos, developed in siliques of heterozygous *abci10-1* and *abci11-1* lines. Empty seed coats in both mutant lines were detected with a high proportion (23-28%, n = 8 siliques per line) which demonstrates an embryo lethal defect for both ho *abci10-1* and *abci11-1* mutants. The arrowhead indicates a green funiculus, which is part of the mother tissue.
