# Supplementary material for: A Novel Prokaryote-Type ECF/ABC Transporter Module in Chloroplast Metal Homeostasis
Source: Front Plant Sci. 2019 Oct 29;10:1264. doi: 10.3389/fpls.2019.01264 (PMC6828968; doi:10.3389/fpls.2019.01264)
Supplement: Supplementary file 6 [file Image_6.pdf]

Figure S6

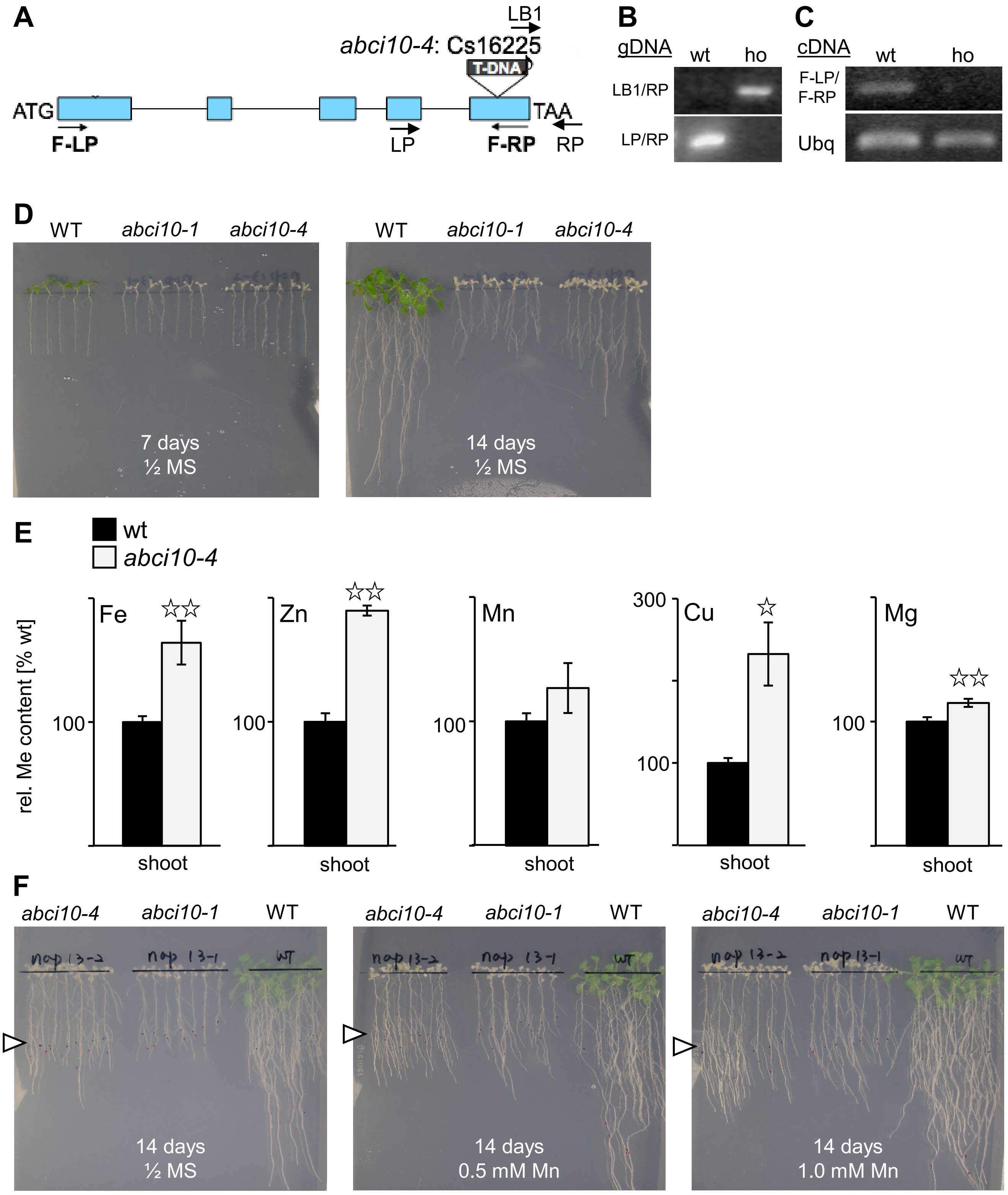
A
LB1
abci10-4: Cs16225
T-DNA
ATG
TAA
F-LP
LP
F-RP
RP
B
gDNA
wt
ho
LB1/RP
LP/RP
C
cDNA
wt
ho
F-LP/
F-RP
Ubq
D
WT
abci10-1
abci10-4
7 days
½ MS
WT
abci10-1
abci10-4
14 days
½ MS
E
wt
abci10-4
rel. Me content [% wt]
Fe
☆☆
100
shoot
Zn
☆☆
100
shoot
Mn
100
shoot
300
Cu
☆
100
shoot
Mg
☆☆
100
shoot
F
abci10-4
abci10-1
WT
14 days
½ MS
abci10-4
abci10-1
WT
14 days
0.5 mM Mn
abci10-4
abci10-1
WT
14 days
1.0 mM Mn

FIGURE S6 | Characterization of the T-DNA mutant *abci10-4.*
(A) The gene *At-ABCI10* (At4g33460) contains 5 exon regions (blue boxes). The T-DNA insertion for the mutant line *abci10-4* (Cs16225) is in the fifth exon. Positions of oligonucleotide primers used for genotyping and RT-PCR are indicated by arrows. Please note that RP is located in the 3'UTR behind the stop codon "TAA". **(B)** Genomic DNA (gDNA) of *abci10-4* was screened by PCR using the oligonucleotide primers LP, RP (specific for *At-ABCI10*) and LB1 (specific for the T-DNA), see **(A)**. The primer combination LB1/RP gave PCR products on homozygous (ho) but not on wild-type (wt) alleles (upper panel), while products of LP/RP in wt were absent on DNA of ho *abci10-4* plants (lower panel). **(C)** RT-PCR on cDNA from wild-type (wt) and segregated homozygous (ho) plants of *abci10-4*. The primer pair F-LP/F-RP, which is specific for *At-ABCI10* (see **(A)**) only in wild-type plants amplifed a product, showing that *abci10-4* homozygous plants are knockouts without mRNA of *At-ABCI10*. A PCR product for Ubiquitin (Ubq) was used as control (lower panel). **(D)** Phenotypes of *abci10-1* (compare **Figures 4, S5, S7**) and *abci10-4* lines (7 and 14-day-old seedlings, grown on ½ MS agar plates). Please note that homozygous (ho) mutants for *abci10-1* and *abci10-4* with T-DNA insertions in exon regions show the characteristic, albino phenotype for the knockout of *At-ABCI10* (compare **Figures 4A, S7**). **(E)** Metal contents (compare **Figure 6**) for iron (Fe), zinc (Zn), manganese (Mn), copper (Cu) and magnesium (Mg) were determined in separated shoot tissue of 15-day-old wild type (wt, black bars) as well as 34-day-old *abci10-4* mutant lines (grey bars). The respective metal content ($n=3 \pm SD$, for Cu $n=2$) is given relative to the level in wt, which was set to 100%. Data points with significant difference to wt according to Student's t-test are indicated by * ($p < 0.05$) and ** ($p < 0.01$). **(F)** Manganese rescue of *abci10-1* and *abci10-4* root growth. After germination for 7 days on ½ MS media, seedlings were transferred to ½ MS supplemented with 0, 0.5 or 1.0 mM Mn. Photos were taken 14 days after transfer. Thus, 21-day-old seedlings are several days younger than for the assay depicted in **Figure S7C**. Black dots and triangles indicate the root lengths directly after the transfer. Please note that *abci10-1* and *abci10-4* in this assay originally were named nap13-1 and nap13-3, respectively as depicted on the plates.
