# Supplementary material for: A Novel Prokaryote-Type ECF/ABC Transporter Module in Chloroplast Metal Homeostasis
Source: Front Plant Sci. 2019 Oct 29;10:1264. doi: 10.3389/fpls.2019.01264 (PMC6828968; doi:10.3389/fpls.2019.01264)
Supplement: Supplementary file 7 [file Image_7.pdf]

Figure S7

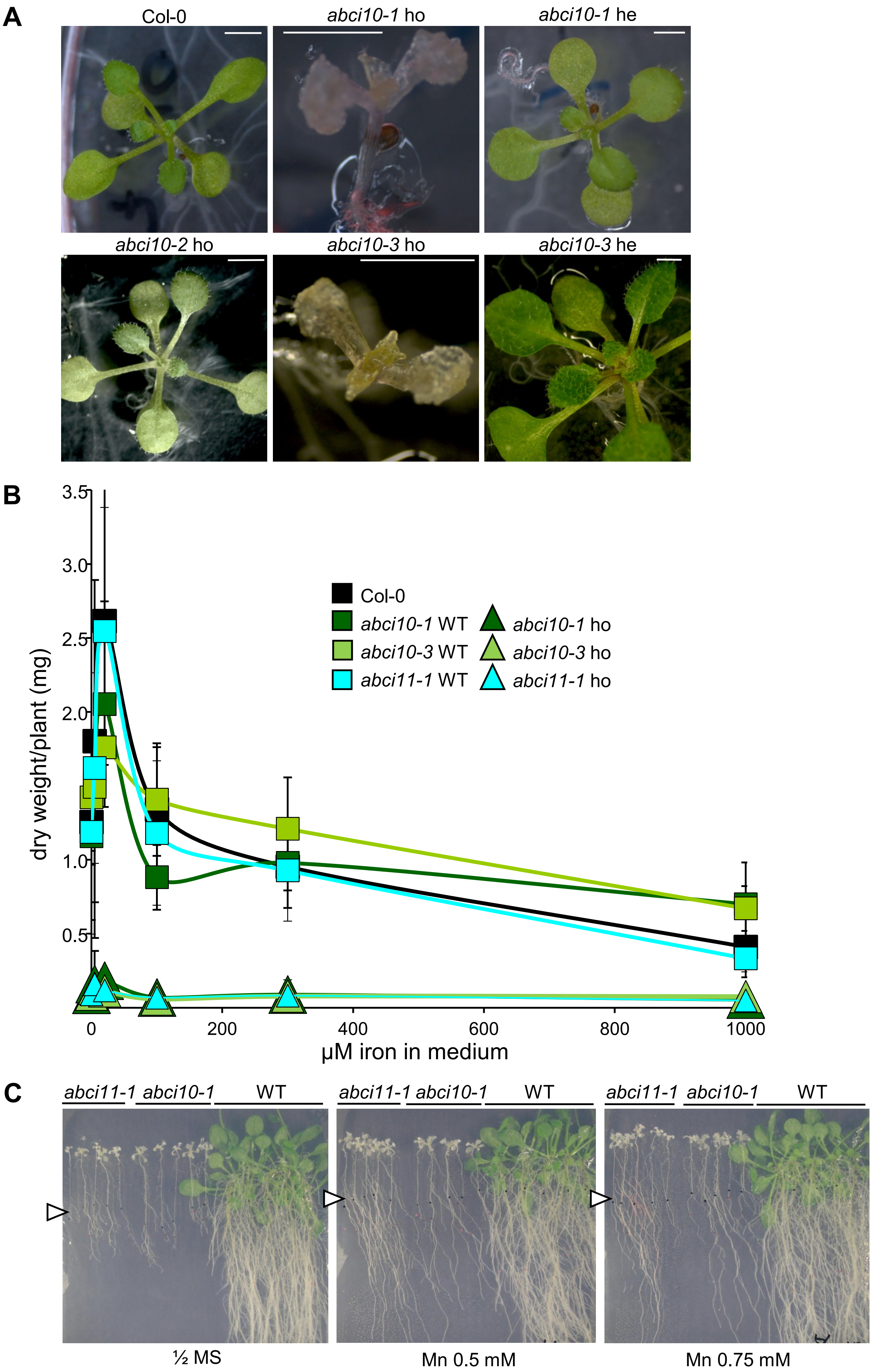
A
Col-0
abci10-1 ho
abci10-1 he
abci10-2 ho
abci10-3 ho
abci10-3 he
B
3.5
3.0
2.5
2.0
1.0
0.5
dry weight/plant (mg)
Col-0
abci10-1 WT
abci10-3 WT
abci11-1 WT
abci10-1 ho
abci10-3 ho
abci11-1 ho
0
200
400
600
800
1000
µM iron in medium
C
abci11-1
abci10-1
WT
½ MS
Mn 0.5 mM
Mn 0.75 mM

FIGURE S7 | The dwarfed phenotype of *abci10, abci11* knockout lines is independent on Fe supply, but short root growth can be resuced by Mn.

(A) Phenotypes of *abci10-1, abci10-2,* and *abci10-3* lines (20-day-old seedlings) as described in **Figure S5**. Please note that homozygous (ho) mutants for *abci10-1* and *abci10-3* with T-DNA insertions in exon regions show the characteristic, albino phenotype for the knockout of *At-ABCI10* (compare **Figure 4A**). The ho line *abci10-2* (T-DNA insertion in the 3' UTR) instead has green wild-type like appearance and therefore can be considered as control, similar to a complemented knockout line. **(B)** Severely reduced dry weight of *abci10* and *abci11* mutants is independent on Fe supplementation. The dry weight of *abci10-1*, *abci10-3*, *abci11-1* homozygous knockout mutants (green, light green, blue triangles), of the corresponding segregated wild-type lines *abci10-1* WT, *abci10-3* WT, *abci11-1* WT as well as of Col-0 (green, light green, blue, black squares) was determined from 3-week-old seedlings grown on ½ MS media, supplemented with 0, 5, 20, 100, 300 and 1000 µM Fe. Data points (n=3-9 ± SD) represent the weight of freeze-dried plants (2-15 sampled individuals per data point) from at least 3 biological replicates, i.e. independently grown on the respective media. **(C)** Manganese rescue of *abci10-1* and *abci11-1* root growth. After germination for 10 (wt) and 13 days (*abci10-1*, *abci11-1*) on ½ MS media, seedlings were transferred to ½ MS, supplemented with 0, 0.5 or 0.75 mM Mn. Photos were taken 14 days after transfer. Please note that 24-day-old wt and 27-day-old mutant seedlings therefore are several days older than plants (21-day-old) for the assay depicted in **Figure S6F**. Black dots and triangles indicated the root lengths directly after the transfer. Other divalent cations (Mg, Fe, Cu, Zn, Co) were not able to rescue root growth (data not shown, compare **(B)** for Fe impact on dry weight), indicating a Mn specific effect.
