# Supplementary material for: A Novel Prokaryote-Type ECF/ABC Transporter Module in Chloroplast Metal Homeostasis
Source: Front Plant Sci. 2019 Oct 29;10:1264. doi: 10.3389/fpls.2019.01264 (PMC6828968; doi:10.3389/fpls.2019.01264)
Supplement: Supplementary file 8 [file Image_8.pdf]

Figure S8

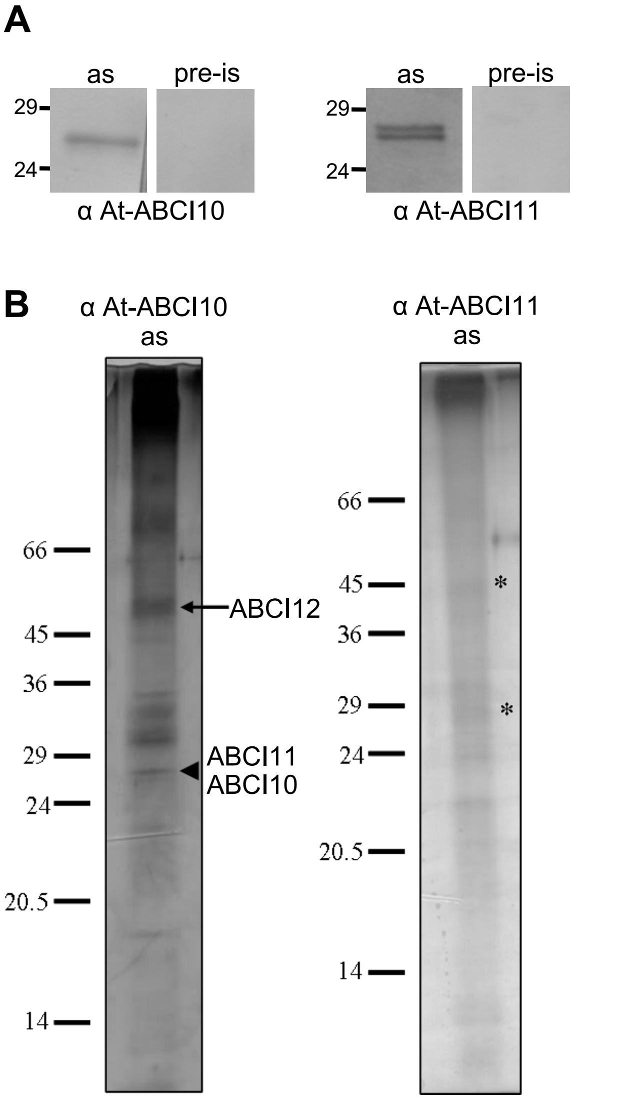

FIGURE S8 | Co-immunoprecipitation assays. Co-immunoprecipitation (co-ip) assays were performed with the antiserum (as) directed against At-ABCI10 (α-At-ABCI10, left panels) or with α-At-ABCI11 (right panels). For control of unspecific interactions, the assay was repeated with the corresponding pre-immune sera (pre-is). **(A)** Immunoblots on elution fractions of co-ip assays with At-ABCI10 as / pre-is (left) and At-ABCI10 as / pre-is (right). The antisera for At-ABCI10 and At-ABCI11 both were able to precipitate At-ABCI10 (27 kDa) and At-ABCI11 (28.5 kDa), respectively, from the solubilized pea chloroplast IE membranes, as indicated by the specific staining in the immunoblots. Please note that α-At-ABCI11 detects a double band between 28-29 kDa as observed before (compare **Figure 3A**). **(B)** Silver stained SDS gels with equal amounts of elution fractions from co-ip assays with At-ABCI10 (left) and At-ABCI11 (right) antisera. Please note that α-At-ABCI10 precipitated more proteins than α-At-ABCI11. The elution fraction of the latter resembled that of the respective pre-is controls. Protein sequencing of the 50 kDa band (arrow) in the α-At-ABCI10 co-ip revelaed peptides of ABCI12, pointing to interaction between ABCI10 and ACBI12 in the chloroplast IE membrane. Further, α-At-ABCI10 precipitated ABCI10 and ACBI11 proteins in a band around 28 kDa (triangle).
