# Supplementary material for: A Novel Prokaryote-Type ECF/ABC Transporter Module in Chloroplast Metal Homeostasis
Source: Front Plant Sci. 2019 Oct 29;10:1264. doi: 10.3389/fpls.2019.01264 (PMC6828968; doi:10.3389/fpls.2019.01264)
Supplement: Supplementary file 9 [file Image_9.pdf]

Figure S9

A At-ABCI10-GFP + AtABCI12-mRFP

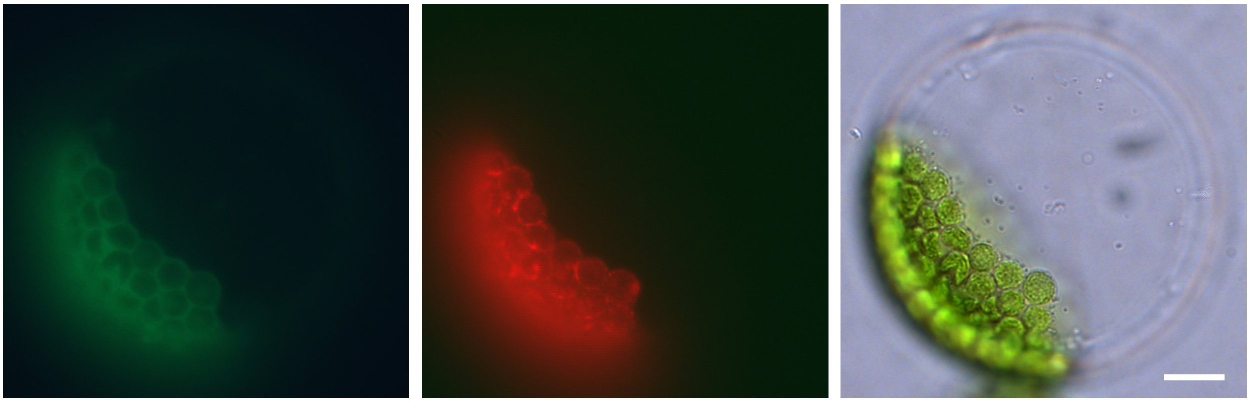

B At-ABCI11-GFP + AtABCI12-mRFP

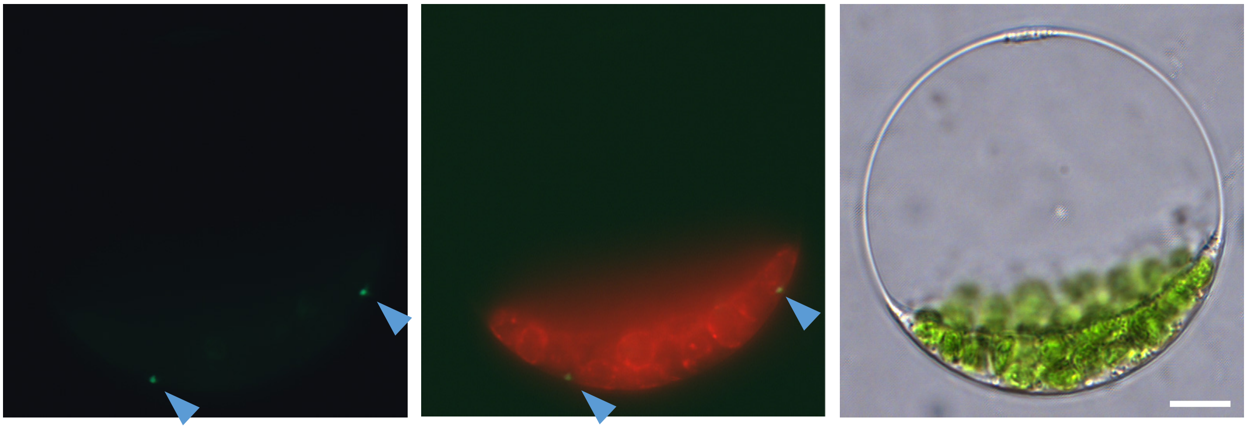

FIGURE S9 | At-ABCI12 in the chloroplast envelope targets At-ABCI10 to the IE membrane. *Arabidopsis* leaf protoplasts were transiently co-transformed with constructs for At-ABCI10-GFP and At-ABCI12-mRFP **(A)** or with At-ABCI11-GFP and At-ABCI12-mRFP **(B).** Images show GFP- and RFP signals (left and middle panel) as well as a bright field overview (right). As observed in **Figure 8D**, co-expression with ABCI12 targets green GFP signals of ABCI10 to the envelope membrane. In contrast, ABCI11-GFP (**(B)**, left image), still stains plastoglobuli-like structures (blue triangles) when co-expressed with ABCI12 and thus shows the same pattern as when transformed as single construct. Scale bar = 10 µm.
