# Supplementary material for: A Novel Prokaryote-Type ECF/ABC Transporter Module in Chloroplast Metal Homeostasis
Source: Front Plant Sci. 2019 Oct 29;10:1264. doi: 10.3389/fpls.2019.01264 (PMC6828968; doi:10.3389/fpls.2019.01264)
Supplement: Supplementary file 10 [file Image_10.pdf]

Figure S10

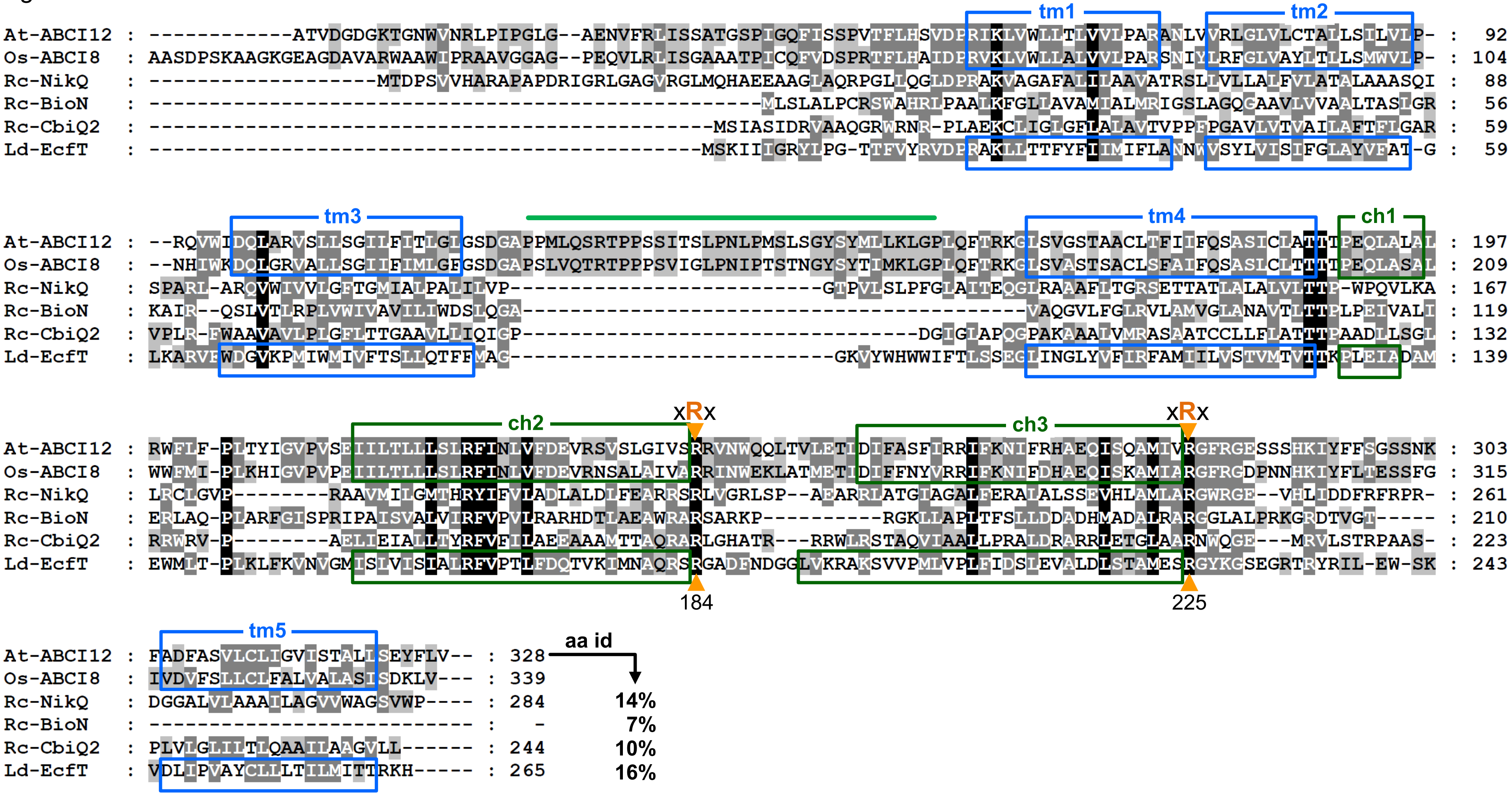

FIGURE S10 | At-ABCI12 is an ECF-type T subunit.

Amino acid sequences of mature *Arabidopsis* At-ABCI12 (Q944H2), Os-ABCI8 (Q6ATY7), and the bacterial ECF transporter A subunits Rc-NikQ (D5AQY7), Rc-BioN (D5ARG9), Rc-CbiQ2 (D5AUZ7) from *Rhodobacter capsulatus* and Ld-EcfT (A0A061BSU4) from *Lactobacillus delbrueckii*. UniProtKB accession numbers are given in brackets, chloroplast targeting peptides were predicted by ChloroP (Emanuelsson et al., 1999). The transmembrane helices tm1-5 (blue boxes) and the coupling helices ch1-3 (green boxes) are depicted according to the structural model of At-ABCI12 (Phyre2; Kelley et al. 2015) and the crystal structure of Ld-EcfT (pdb database 5D7T; Swier et al. 2016). Please note that BioN only contains 4 tm helices. The two conserved X-R-X motifs at the end of ch2 and ch3 (R_{184}, R_{225} of EcfT), which are responsible for binding to the ATPase AA dimer (compare **Figure 1**) are indicated by orange arrow heads. The peptide stretch between tm3 and tm4, which is specific for plant and cyanobacterial T proteins (compare Eitinger et al., 2011) is highlighted by a green line.
