# Supplementary material for: A Novel Prokaryote-Type ECF/ABC Transporter Module in Chloroplast Metal Homeostasis
Source: Front Plant Sci. 2019 Oct 29;10:1264. doi: 10.3389/fpls.2019.01264 (PMC6828968; doi:10.3389/fpls.2019.01264)
Supplement: Supplementary file 11 [file Image_11.pdf]

Figure S11

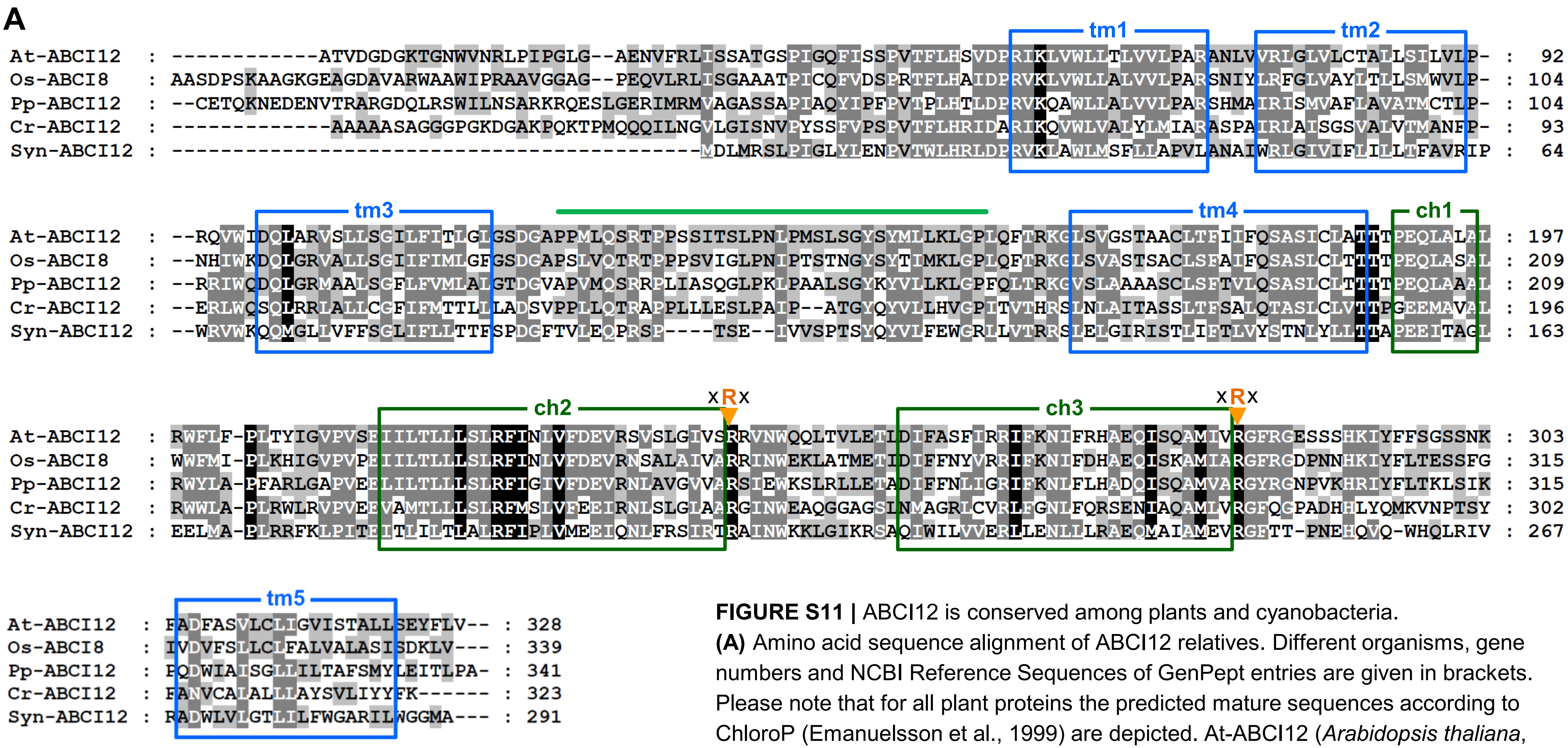

B

	At	Os	Pp	Cr	Syn
At-ABCI12	100%				
Os-ABCI8	59%	100%			
Pp-ABCI12	46%	48%	100%		
Cr-ABCI12	36%	35%	36%	100%	
Syn-ABCI12	28%	29%	28%	30%	100%

FIGURE S11 | ABCI12 is conserved among plants and cyanobacteria. **(A)** Amino acid sequence alignment of ABCI12 relatives. Different organisms, gene numbers and NCBI Reference Sequences of GenPept entries are given in brackets. Please note that for all plant proteins the predicted mature sequences according to ChloroP (Emanuelsson et al., 1999) are depicted. At-ABCI12 (*Arabidopsis thaliana*, At3g21580, NP_566688), Os-ABCI8 (*Oryza sativa [japonica]*, Os05g33230, XP_015639696), Pp-ABCI12 (*Physocomitrella patens*, XP_024398704), Cr-ABCI12 (*Chlamydomonas reinhardtii*, PNW75614), Syn-CBY (*Synechocystis PCC6803*, gene slr1978, WP_010872838). The transmembrane helices tm1-5 (blue boxes) and the coupling helices ch1-3 (green boxes) are depicted according to the structural model of At-ABCI12 (Phyre2; Kelley et al., 2015) The two conserved X-R-X motifs at the end of ch2 and ch3, which most likely are responsible for binding to the ATPase AA dimer (compare **Figures 1, S10**) are indicated by orange arrow heads. The peptide stretch between tm3 and tm4, which is specific for plant and cyanobacterial T proteins (compare **Figure S10**; Eitinger et al., 2011) is highlighted by a green line. **(B)** Amino acid identities in % of proteins depicted in **(A)**.
